# Pyra-metho-carnil disrupts cancer cell proteostasis and induces apoptosis by binding to KDEL receptors

**DOI:** 10.1038/s41598-026-45604-z

**Published:** 2026-03-26

**Authors:** Kazumasa Yoshida, Kensuke Nishi, Takanori Kitaguchi, Taichi Matsumoto, Gen Maruta, Hisanori Maeoka, Shuhei Ishikura, Senji Shirasawa, Toshiyuki Tsunoda

**Affiliations:** 1 Department of Cell Biology, Faculty of Medicine, Fukuoka University, 7-45-1 Nanakuma, Jonan-Ku, Fukuoka, 814-0180 Japan; 2 Research Institute for Advanced Molecular Medicine, Fukuoka University, 7-45-1 Nanakuma, Jonan-Ku, Fukuoka, 814-0180 Japan; 3 Oral Medicine Research Center, Fukuoka Dental College, 2-15-1 Tamura, Sawara-Ku, Fukuoka, 814-0193 Japan

**Keywords:** Endoplasmic reticulum stress, Unfolded protein response, CHOP, KDEL receptors, Proteostatic disruption, Aggresome

## Abstract

We previously demonstrated that the carboline derivative pyra-metho-carnil (PMC) inhibits the proliferation of multiple cancer cell lines. In this study, we investigated the underlying mechanisms by screening for PMC-interacting proteins and testing for effects on protein function. Affinity purification using PMC-immobilized beads followed by gel electrophoresis and mass spectrometry revealed that PMC binds to KDEL receptors (KDELRs), 7-transmembrane proteins regulating the retrieval (retrograde transport) of endoplasmic reticulum (ER)-resident proteins with a C-terminal Lys-Asp-Glu-Lue (KDEL) motif, from the Golgi apparatus. Furthermore, using the Fluoppi system for live-cell imaging, we confirmed that PMC disrupts the interaction between KDELR1 and its ligand BiP. Treatment of HCT116 cells with PMC also disrupted the Golgi localization of GFP-tagged KDELR proteins and promoted the accumulation of misfolded protein aggregates. Indeed, PMC triggered the unfolded protein response (UPR), enhanced CHOP-mediated caspase activation, and reduced viable cell number, suggesting that KDELR inhibition induces proteostatic disruption and leads to apoptotic cell death. Furthermore, PMC sensitized cells to ER stress induced by thapsigargin. Knockdown of KDELR using small interfering (si)RNAs also activated the UPR and suppressed cancer cell proliferation. These findings suggest that PMC inhibits cancer cell proliferation and survival by impairing KDELR function and inducing proteostatic disruption. We propose that KDELR inhibitor drugs may be useful as primary or adjuvant therapeutics for cancer.

**Supplementary Information:**

The online version contains supplementary material available at 10.1038/s41598-026-45604-z.

## Introduction

Pharmaceutical interventions that target signaling pathways and metabolic abnormalities required for cancer cell proliferation and tumor growth are emerging as highly effective treatments without the systemic toxicity of conventional chemotherapies^1,2^. However, many of these drugs are prone to evolving resistance or have adverse side effects, and there remain many cancer-related pathways without specific chemical modulators^3^. It is therefore essential to develop novel agents that exploit known and unexplored molecular vulnerabilities in cancer cells. We previously identified pyra-metho-carnil (PMC, so named due to the presence of pyrazole, methoxyphenyl, and β-carboline structures) from a natural compound library that inhibits the spheroid growth of multiple tumor cell lines irrespective of tissue type, driver gene mutation, and drug resistance profile, suggesting that PMC may target important signaling processes common to multiple malignancies^4^. Indeed, we also demonstrated that PMC downregulates aerobic glycolysis of cancer cells and suppresses the differentiation of macrophages that promote cancer growth^5,6^. However, the molecular mechanisms underlying the suppression of cancer growth by PMC remain obscure.

In this study, we aimed to identify the target molecules of PMC by affinity purification of cell lysate proteins using PMC-immobilized beads and found that PMC specifically binds to KDEL receptors (KDELRs), which function as part of the Golgi-to-endoplasmic reticulum (ER) vesicle retrograde transport system to retrieve ER-resident proteins and maintain ER proteostasis^7–10^. We further found that PMC altered the normal subcellular localization of KDELRs and triggered intense activation of the unfolded protein response (UPR), followed by cell death. These findings suggest that the cytotoxic effects of PMC involve ER stress induced by the attenuation of KDELR function. Taken together, our results support KDELR as a novel therapeutic target molecule for the proteostatic disruption and ensuing destruction of cancer cells.

## Results

### Identification of KDELRs as PMC-binding proteins

**Fig. 1:**
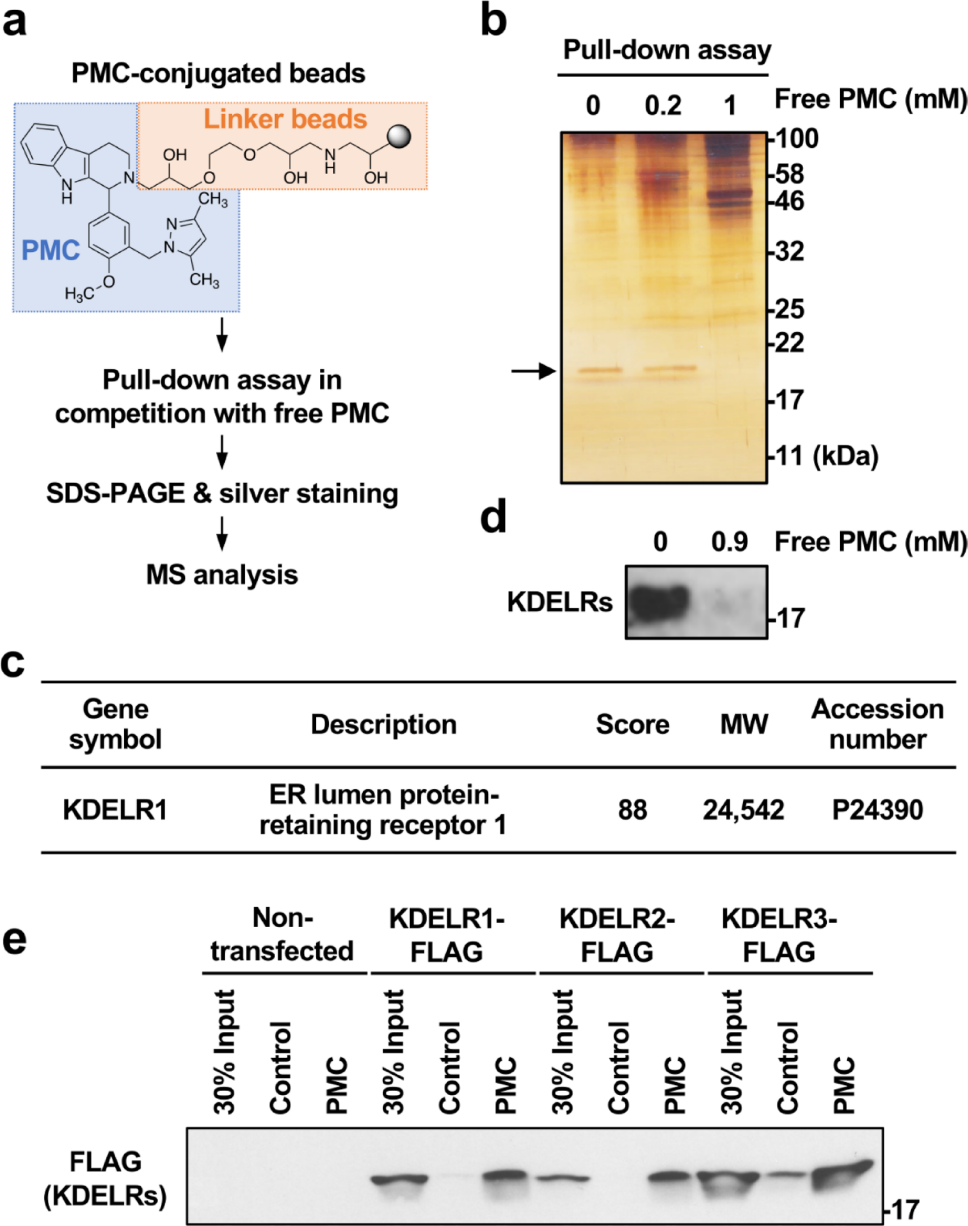
Identification of KDEL receptors as candidate PMC targets by pull-down analysis of HKe3-mutKRAS cell extracts using PMC-conjugated beads. (**a**) Flowchart of the pull-down analysis for identifying PMC-binding proteins. (**b** and **d**) SDS-PAGE separation of proteins pulled down by PMC-conjugated beads from HKe3-mutKRAS cell extracts supplemented with the indicated concentrations of free PMC, followed by silver staining (**b**) or immunoblotting with an anti-KDELR antibody (**d**). The silver staining experiment was performed once to obtain material for MS analysis (**b**), whereas the immunoblotting data are representative of two independent experiments (**d**). (**c**) Information on the protein identified by MS analysis of a gel slice corresponding to the position where the visible band (arrow) was eliminated by inclusion of free PMC. The gene symbol, description, Mascot score, molecular weight, and UniProt accession number are shown. (**e**) Pull-down analysis of FLAG-tagged KDEL receptors KDELR1–3 in HCT116 cells transiently transfected with the indicated expression vector using non-conjugated linker beads (control) or PMC-conjugated beads (PMC). Data are representative of two independent experiments.

To screen for PMC-binding proteins, we prepared PMC-conjugated magnetic ferrite-glycidyl methacrylate (FG) beads via an epoxy linker and conducted a pull-down assay using extracts of HKe3-mutKRAS cells, the cell line used in the original screening for PMC^4^ (Fig. 1a). HKe3-mutKRAS cells were derived in a stepwise manner from the HCT116 human colorectal cancer cell line through establishment of HKe3 cells, a clonal cell line in which the endogenous mutated KRAS (mutKRAS) gene was disrupted^11,12^. In HKe3-mutKRAS cells, the ectopic mutKRAS gene was stably reexpressed to investigate inhibitors that target the mutKRAS-mediated signaling pathway^4,12^. Bound proteins on the PMC-conjugated beads were separated by SDS-PAGE, followed by silver staining (Fig. 1b). To control for nonspecific binding, proteins pulled down as above were compared to proteins bound to the PMC-conjugated beads in the presence of excess free PMC. Addition of free PMC as a competitor resulted in the disappearance of a visible protein band with an apparent molecular mass of approximately 20 kDa (Fig. 1b, arrow). Mass spectrometricanalysis of the gel slice containing this protein band identified KDELR1 (Fig. 1c).

Mammals express three highly homologous KDELR isoforms, KDELR1–3, all of which function to regulate the retrograde transport of ER-resident proteins from downstream Golgi compartments^7,13–16^. We confirmed the binding of KDELRs to PMC through a pull-down assay, followed by immunoblotting with an anti-KDELR monoclonal antibody (KR-10) that recognizes all three isoforms^17^ (Fig. 1d). In addition, the interaction of each isoform with PMC was validated through pull-down analysis of HCT116 cells transiently expressing FLAG-tagged KDELR1, KDELR2, or KDELR3 (Fig. 1e). Collectively, these results indicate that PMC binds to all three KDELRs.

### PMC promotes mislocalization of KDELRs and accumulation of protein aggregates

**Fig. 2:**
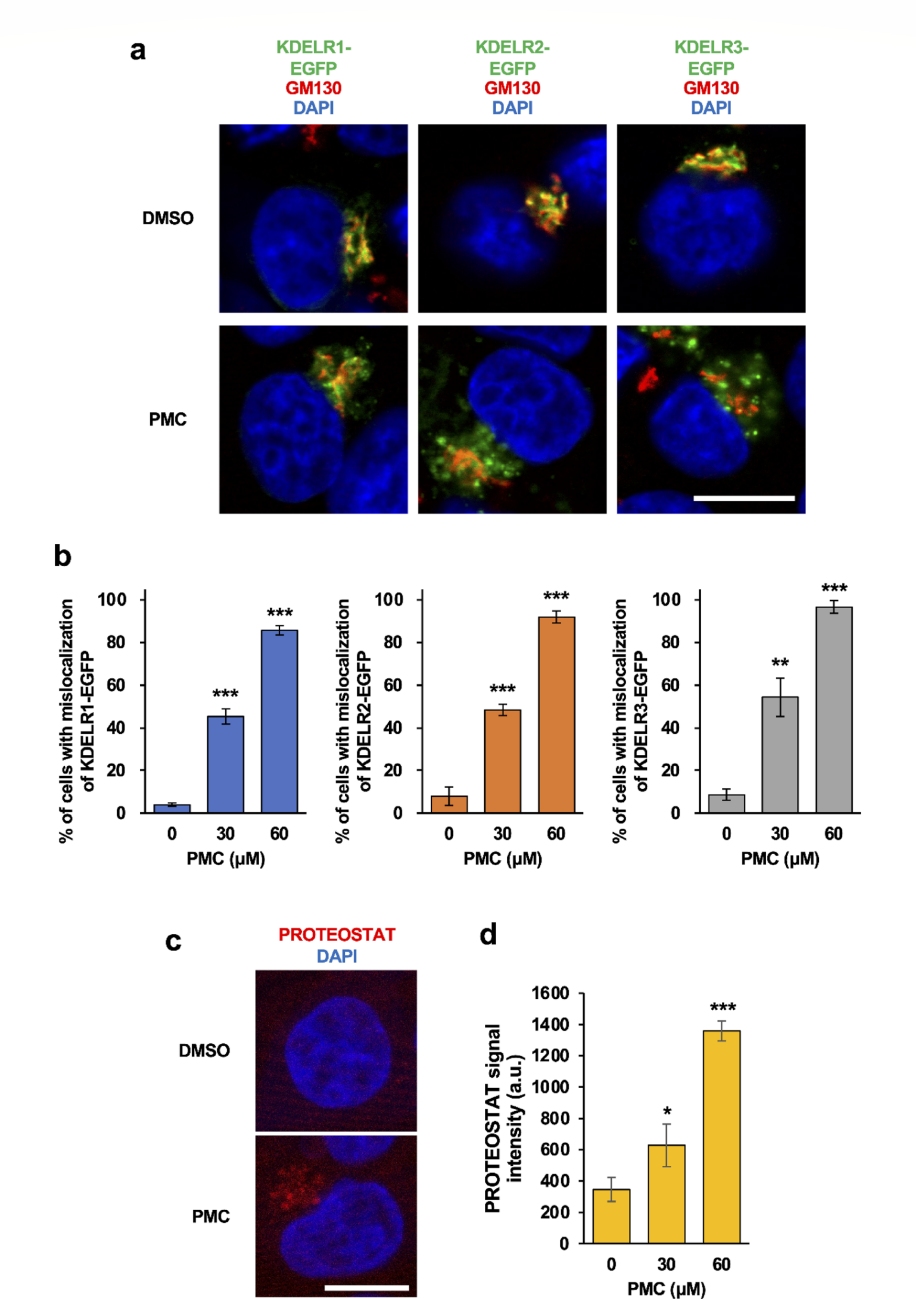
PMC induces mislocalization of KDELR-EGFPs and accumulation of protein aggregates. (**a**) Immunofluorescence images of HCT116 cells transiently expressing KDELR1-EGFP, KDELR2-EGFP, or KDELR3-EGFP (all green) and immunostained for the cis-Golgi marker GM130 (red). Nuclear DNA was counterstained with DAPI (blue). Cells were treated with DMSO as a control or 60 μM PMC for 24 h. (**b**) Proportion of cells (%) demonstrating mislocalization (non-Golgi distribution) of KDELR1-EGFP, KDELR2-EGFP, or KDELR3-EGFP. Results derived from images described in (**a**). (**c** and **d**) Fluorescence images (**c**) and quantification (**d**) of HCT116 cells treated with DMSO or 60 μM PMC for 6 h and stained for the protein aggregate dye PROTEOSTAT and counterstained with DAPI. (**a**, **c**) Scale bar, 10 μm. (**b**, **d**) Data represent mean ± standard deviation (SD) of three independent experiments. *p < 0.05; **p < 0.01; ***p < 0.001 vs. DMSO-treated cells by Student’s t-test.

The KDELRs are seven-transmembrane receptor proteins that reside primarily in the cis-Golgi network, where they facilitate retrograde vesicle transport from the Golgi to ER upon binding to Lys-Asp-Glu-Leu (KDEL) or variant tetrapeptide sequences of primarily soluble ER-resident molecular chaperone proteins^7,8,18^. This process drives the recycling of soluble ER proteins containing KDEL sequences back into the ER lumen to maintain ER integrity and the homeostasis of membrane and secreted proteins^8,19^. To investigate the effects of PMC on KDELR function, we first examined their subcellular distribution by expressing enhanced green fluorescent protein (EGFP)-tagged KDELRs (KDELR1-EGFP, KDELR2-EGFP, and KDELR3-EGFP) in HCT116 cells (Fig. 2a, b) and treating these cells with vehicle or PMC. In DMSO-treated control cells, KDELR-EGFPs were primarily located in the cis-Golgi as indicated by colocalization with the cis-Golgi marker protein GM130, a distribution consistent with previous studies on KDELRs. This observation suggests that EGFP tagging does not interfere with the normal cis-Golgi localization of KDELRs. In contrast, all three KDELR-EGFPs were localized outside the cis-Golgi in PMC-treated cells, and quantification showed that this mislocalization was PMC dose-dependent, with approximately 90% of cells exhibiting aberrant localization at 60 μM PMC (Fig. 2b). In addition, PMC altered the localization of KDELR-EGFPs in HeLa cells, a line derived from cervical cancer cells (Supplementary Fig. S1). To examine whether PMC affects the interaction between KDELRs and the KDEL-containing ER chaperone protein BiP, we employed the Fluoppi system, which enables the visualization of protein–protein interactions in living cells^20,21^. We established HCT116 cells expressing KDELR1-hAG alone (HCT116K) and those co-expressing KDELR1-hAG and Ash-BiP (HCT116KB). In HCT116K cells, several intense Fluoppi puncta were consistently observed throughout the observation period following PMC treatment. These signals were independent of Ash-BiP and were therefore considered to be false positives (Supplementary Movie S1A; White arrows). In contrast, PMC treatment caused a time-dependent disappearance of Fluoppi puncta in HCT116KB cells (Supplementary Movie S1B). These results indicate that PMC disrupts the interaction between KDELR1 and BiP in living cells.

Given that KDELRs are essential for maintaining ER functional integrity (e.g., returning molecular chaperones to the ER lumen), impaired localization of KDELRs may result in defective protein folding and trafficking from the ER. To investigate these potential effects, we monitored protein aggregation in the presence and absence of PMC treatment using PROTEOSTAT dye, which specifically visualizes misfolded and aggregated proteins^22^. As predicted, PMC dose-dependently induced protein aggregation (Fig. 2c, d), implying disruption of KDELR function and protein homeostasis (proteostasis).

### PMC triggers the UPR and induces CHOP-mediated cell death

**Fig. 3:**
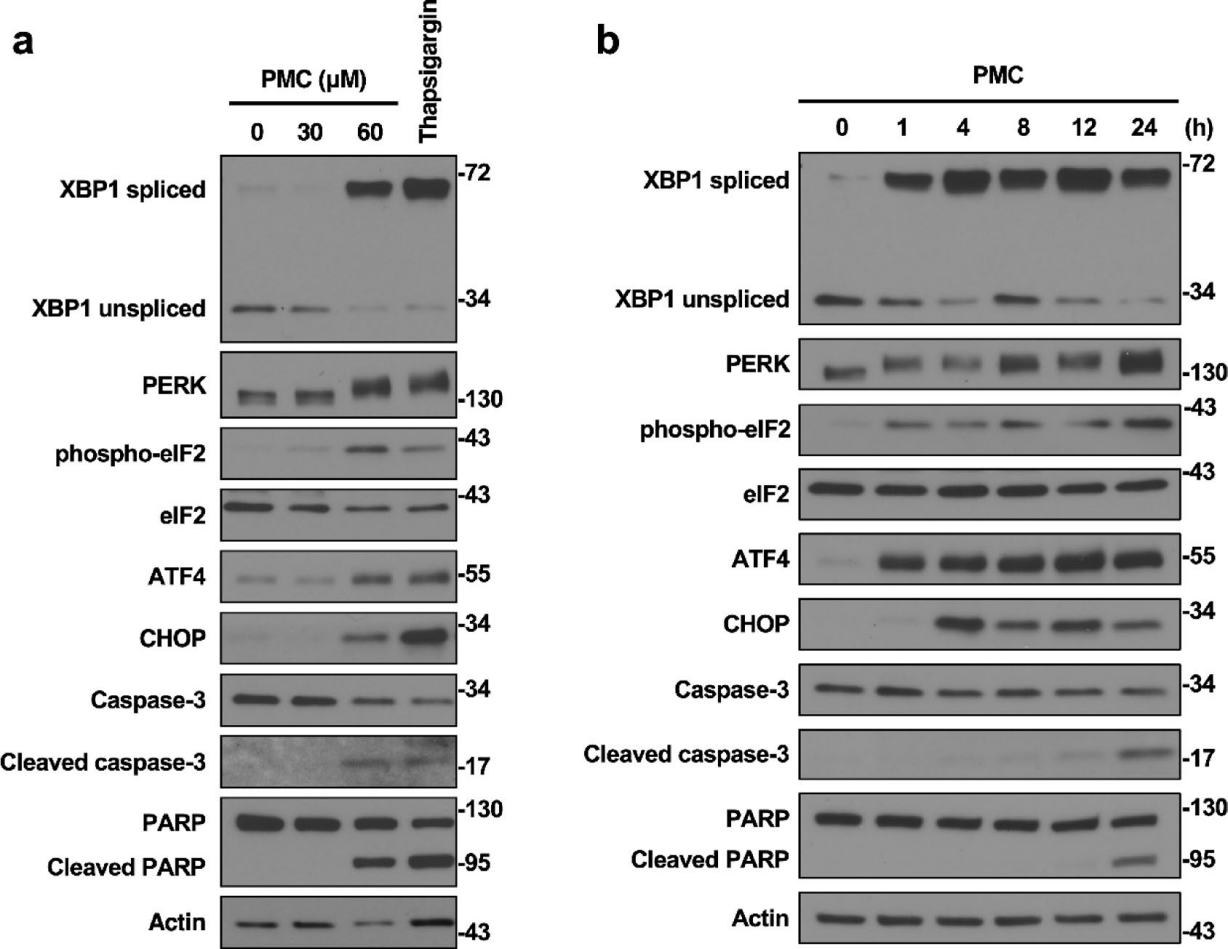
PMC activates the unfolded protein response. (**a** and **b**) Immunoblotting analyses of HCT116 cells treated with increasing concentrations of PMC or 0.05 μM thapsigargin for 24 h (**a**) or with 60 μM PMC for the indicated durations (**b**). Actin was used as a gel loading control. Data are representative of three independent experiments.

An imbalance between protein folding demand and ER capacity results in the accumulation of unfolded proteins in the ER lumen, which creates an unfavorable condition known as ER stress^23,24^. In response to ER stress, adaptive UPR pathways are activated to restore proteostasis by regulating the rate of protein synthesis, protein-folding capacity, and the degradation of unfolded protein^25–28^. However, when ER stress is severe and prolonged, and proteostasis cannot be reestablished by these adaptive UPR pathways, terminal UPR pathways are activated to remove stressed cells by inducing apoptotic cell death^29,30^. Here, we hypothesized that the documented cytotoxic effect of PMC results from disruption of KDELR-regulated trafficking, ER stress, activation of the terminal UPR, and ensuing apoptosis. To test this hypothesis, we first examined whether PMC activates the adaptive and terminal UPR pathways by monitoring the expression levels of key marker proteins in PMC-treated and control HCT116 cells by immunoblotting. Treatment of HCT116 cells with 60 μM PMC markedly elevated expression of XBP1 spliced form (XBP1s), ATF4, and CHOP, and the phosphorylation levels of PERK and eIF2α (as evidenced by a mobility shift of the protein band and phosphorylation-specific antibodies, respectively), indicating activation of the UPR (Fig. 3). Moreover, treatment with 60 μM PMC increased the expression of cleaved (activated) caspase-3, an apoptotic effector protease, and the cleaved form of PARP, a substrate of caspase-3, indicating activation of the terminal UPR-linked apoptotic pathway. In contrast, 30 μM PMC treatment did not influence UPR protein expression levels as assessed by immunoblotting (Fig. 3a), suggesting that ER stress levels may be below the detection threshold at this concentration (Fig. 3a). Time-course experiments on cells treated with 60 μM PMC showed that the expression of XBP1s and ATF4, along with the phosphorylation of PERK and elF2a, significantly increased after only 1 h, while CHOP expression was increased after 4 h of PMC treatment. Activation of these UPR components persisted after 24 h (Fig. 3b), accompanied by elevated cleaved caspase-3 and cleaved PARP expression levels.

**Fig. 4:**
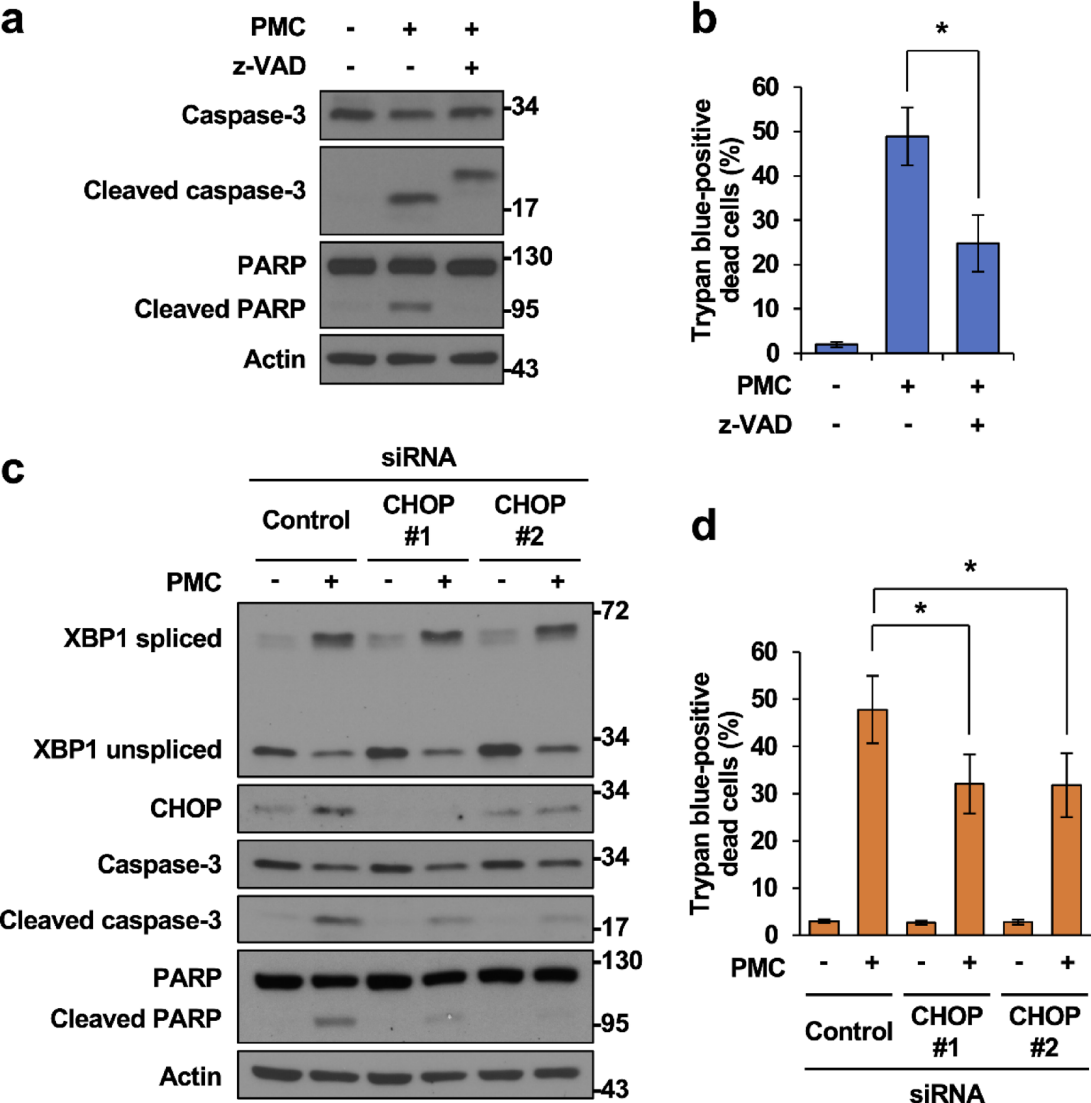
Inhibition of caspase activity and knockdown of CHOP decreases PMC-induced cell death. (**a** and **b**) Immunoblotting analysis (**a**) and proportion of dead HCT116 cells (%) as assessed by trypan blue staining (**b**) following treatment with 60 μM PMC with or without the caspase inhibitor z-VAD-fmk (10 μM) for 24 h. (**c** and **d**) Immunoblotting analysis (**c**) and proportion of dead HCT116 cells (**d**) following transfection with control or CHOP-targeting siRNAs for 24 h and ensuing treatment with 60 μM PMC or DMSO for an additional 24 h. (**a**, **c**) Data are representative of three independent experiments. (**b**, **d**) Data represent mean ± SD of three independent experiments. *p < 0.05 vs. control by Student’s t-test.

Next, we examined whether caspase activation by PMC leads to HCT116 cell death. As expected, cotreatment with the broad-spectrum caspase inhibitor z-VAD-fmk abolished caspase-3 activation (Fig. 4a) and enhanced the exclusion of trypan blue dye by PMC-treated cells (Fig. 4b). We noted an additional higher molecular weight band recognized by the anti-cleaved caspase-3 antibody in the z-VAD-fmk–treated samples (Fig. 4a; uncropped blot in Supplementary Fig. 4A). This band may represent a partially processed caspase-3 species. Such residual processing under z-VAD-fmk treatment could reflect incomplete inhibition or cleavage by alternative proteases. Importantly, downstream PARP cleavage was markedly reduced by z-VAD-fmk (Fig. 4a), consistent with substantial inhibition of caspase activity despite the presence of this band. Collectively, these results suggest that PMC promotes cell death, at least in part, through caspase-mediated apoptosis. To further support this notion, we examined if modulating expression of the UPR-induced transcription factor CHOP alters the PMC response. Knockdown of CHOP expression by transient transfection of a targeted siRNA inhibited PMC-induced activation of caspase-3 and cleavage of PARP but did not alter XBP1s expression (Fig. 4c). Concurrently, CHOP knockdown partially mitigated PMC-induced cell death, as evidenced by the trypan blue exclusion assay, suggesting that CHOP-mediated UPR contributes to cell death in PMC-treated HCT116 cells, although other mechanisms are also involved (Fig. 4d).

### PMC enhances cellular sensitivity to chemically induced ER stress

**Fig. 5:**
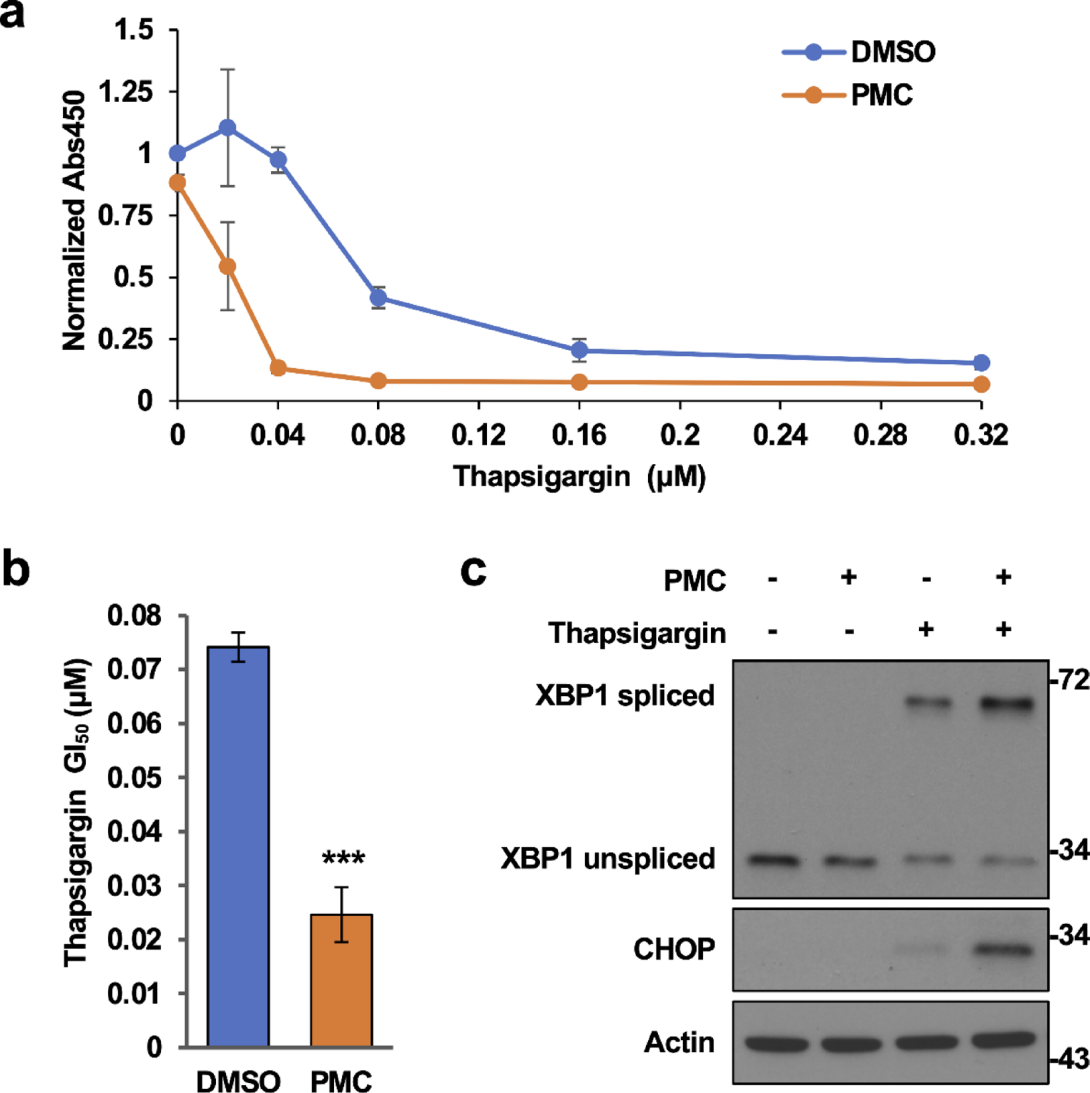
PMC enhances thapsigargin-induced suppression of cell proliferation. (**a** and **b**) Thapsigargin dose–response curves (**a**) and estimated half-maximal thapsigargin concentration for inhibition (GI_50_) (**b**) of HCT116 cell proliferation in the presence of 10 μM PMC or DMSO (control) as determined by the CCK-8 viable cell counting assay. Cells were treated with the indicated drug combination for 48 h. The absorbance at 450 nm (Abs450), indicative of viable cell number was normalized to that of DMSO-treated cells not exposed to thapsigargin. Data represent the mean ± SD of three independent experiments. ***p < 0.001 vs. DMSO-treated control cells by Student’s t-test. (**c**) Immunoblotting analysis of XBP1 and CHOP in HCT116 cells treated with 10 μM PMC and 0.03 μM thapsigargin for 24 h. Actin was used as a gel loading control. Data are representative of three independent experiments.

To further support the contribution of ER stress to these PMC-induced effects on cancer cells, we examined whether PMC influences the sensitivity of HCT116 cells to thapsigargin, a sarcoplasmic/endoplasmic reticulum calcium ATPase (SERCA) blocker widely used to induce ER stress^31^ (Fig. 5a). Indeed, treatment of HCT116 cells with 10 μM PMC, a nontoxic concentration when used alone, sensitized cells to thapsigargin-induced suppression of cell proliferation as indicated by a reduction in GI_50_ (the concentration yielding 50% inhibition of growth) from 0.074 to 0.025 μM (Fig. 5b). In addition, immunoblotting analysis demonstrated that low-dose PMC increased the expression levels of XBP1s and CHOP in the presence of thapsigargin, consistent with a marked enhancement in ER stress sensitivity (Fig. 5c and Supplementary Fig. S2).

### KDELR knockdown induces UPR activation and suppresses cell growth

**Fig. 6:**
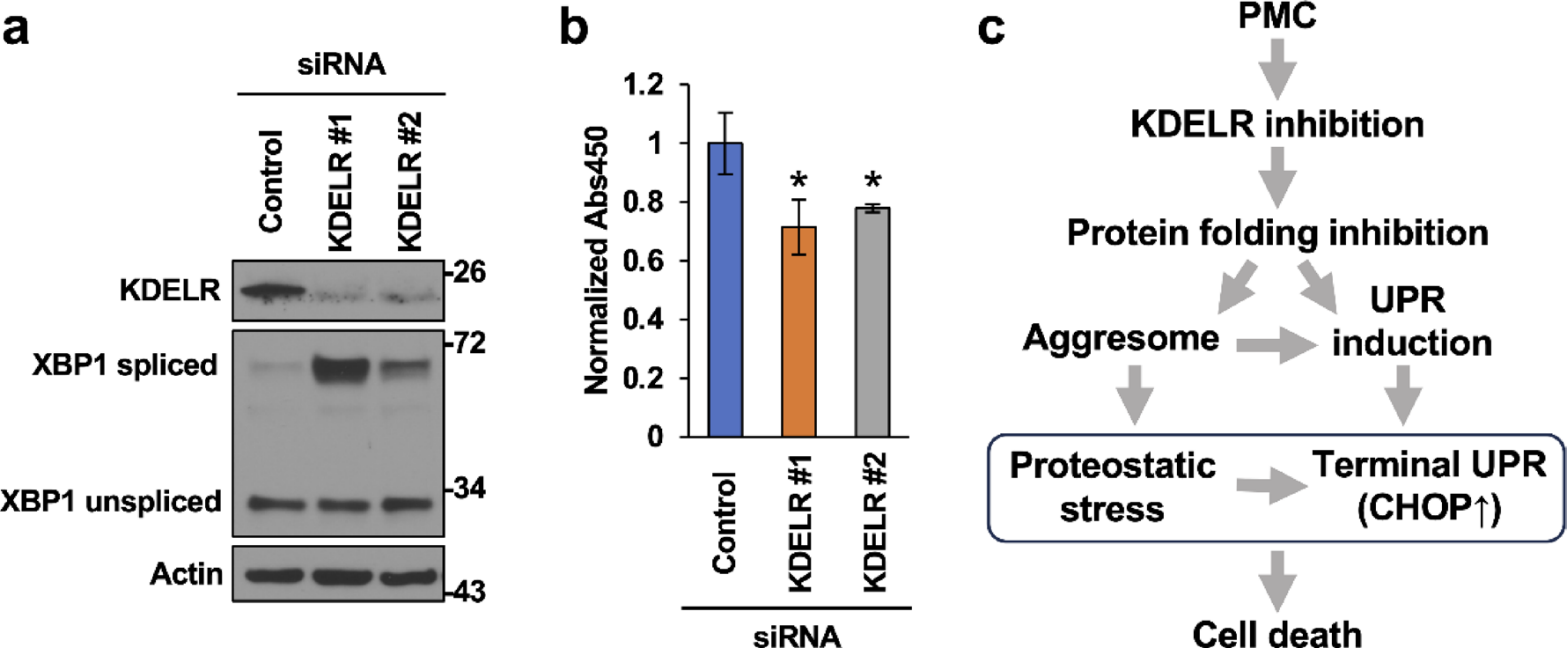
KDELR knockdown induces XBP1 activation and suppresses cell proliferation. (**a**) Immunoblotting analysis of KDELR and XBP1 in HCT116 cells transfected for 48 h with siRNAs targeting KDELRs. Actin was used as a loading control. Data are representative of three independent experiments. (**b**) Proliferation of HCT116 cells transfected for 72 h with siRNAs targeting KDELRs as determined using the CCK-8 viable cell counting assay. The Abs450 was normalized to that of control siRNA-transfected cells. The data represent the mean ± standard deviation (SD) of three independent experiments. *p < 0.05 vs. cells transfected with control siRNA by Student’s t-test. (**c**) Schematic representation of the pathway from PMC exposure to cell death through proteostatic stress. This diagram illustrates how KDELR inhibition leads to impaired protein folding, followed by aggresome formation and UPR induction. These pathways converge to exacerbate proteostatic stress, resulting in terminal UPR characterized by CHOP upregulation and ultimately leading to programmed cell death.

Finally, to provide additional support for the contribution of KDELR binding to PMC-induced ER stress and cancer cell death, we examined if KDELR knockdown alone by transient transfection of targeted siRNA mixtures against all three isoforms (Fig. 6) mimics the effects of PMC. Indeed, knockdown of KDELRs enhanced XBP1s expression and suppressed cell proliferation rate, consistent with our hypothesis that PMC impairs KDELR function, induces harmful ER stress, and consequently inhibits cancer cell proliferation and survival.

## Discussion

The ER is the primary intracellular compartment for the folding and posttranslational modification of membrane-bound and secreted proteins. Cancer cells require enhanced protein synthesis to sustain rapid proliferation and elevated migratory capacity, so mechanisms serving to maintain ER proteostasis are critical for tumor growth, progression, and metastasis, while disrupting these processes may be a novel therapeutic strategy^27,32–35^. However, the development of molecular-targeted therapies that exacerbate proteostasis disruption is still in its infancy. In this study, we identified KDELRs, proteins critical for the maintenance of proteostasis, as target molecules for the novel anticancer agent PMC (Fig. 1). Treatment with PMC resulted in the mislocalization of KDELRs away from the cis-Golgi region of HCT116 and HeLa cells (Fig. 2 and Supplementary Fig. S1), promoted protein aggregation, UPR activation, and CHOP-mediated cell death, and dramatically increased cellular sensitivity to an ER stress inducer (Figs. 2, 3, 4, 5). These findings suggest that the cytotoxicity of PMC involves proteostatic disruption resulting from inhibition of KDELR function. To our knowledge, this is the first report of a small-molecule compound that can interfere with the function of KDELRs, leading to UPR-regulated cell death signaling. We propose that KDELRs are novel druggable targets for cancer therapy.

Genetic perturbations of KDELR have been reported to increase the escape of ER-resident proteins and induce ER stress^17,19,36–39^. Consistent with these reports, KDELR knockdown in HCT116 cells activated the UPR (Fig. 6). While the expression of adaptive UPR genes facilitates cell survival under stress conditions, the activation of terminal UPR pathways under severe ER stress triggers apoptosis^25–30^, and PMC treatment substantially induced both UPR and cell death (apoptotic) markers (Figs. 3 and 4). Specifically, PMC treatment induced IRE1α-dependent mRNA splicing of XBP1 (Fig. 3), which encodes a transcriptional activator of UPR target genes^26,27,35^, and activated the PERK-eIF2α-ATF4-CHOP axis (Fig. 3), which promotes cell death through several pathways including mitochondrial and death receptor-mediated apoptosis pathways^29,30,40,41^. In addition to PERK, IRE1α and activated transcription factor 6 (ATF6) also contribute to CHOP expression^34,40^. Elevated expression levels of XBP1s and ATF4 were observed within 1 h of PMC treatment, suggesting rapid activation of the UPR. In contrast, siRNA-mediated CHOP knockdown attenuated PMC-induced cell death without affecting XBP1 splicing (Fig. 4), suggesting that CHOP acts downstream of XBP1s and ATF4, as expected. Figure 6c outlines the pathway leading from PMC exposure to cell death via disruption of proteostasis. The cascade begins with KDELR inhibition, which subsequently hampers protein folding in the ER. This inhibition branches into two parallel processes: aggresome formation and UPR induction. Both pathways converge, resulting in heightened proteostatic stress. Prolonged stress escalates into a terminal UPR, CHOP upregulation, and ultimately programmed cell death. In the current study, however, PMC-induced cell death was not completely suppressed by CHOP knockdown or by the pan-caspase inhibitor z-VAD-fmk (Fig. 4), indicating that additional CHOP- and caspase-independent pathways contribute to cell death. Consistent with this notion, it has been reported that CHOP is not always indispensable for cell death resulting from ER stress^23,40,42^. Thus, PMC may induce different modes of cell death, including autophagy, ferroptosis, necroptosis, and (or) pyroptosis, which are reported to be induced by ER stress^29,43^. In addition, the accumulation of unfolded proteins results in the formation of toxic aggregates that impair normal cellular function and induce cell death^27,44^.

In addition to their role in retrograde transport, KDELRs are increasingly recognized as regulators of diverse cellular signaling pathways^45^. For example, binding of KDEL-sequence-containing ligands can trigger G-protein-mediated activation of downstream effectors, including protein kinase A, Src family tyrosine kinases, and trafficking regulators Rab1a/Rab3a. KDELR-activated signaling pathways regulate secretion and vesicular trafficking, and influence cell proliferation, survival, and migration. Beyond UPR activation, PMC-induced mislocalization of KDELRs may perturb these cascades, thereby contributing to the anticancer effects of PMC. As our study focused on UPR activation, investigating whether PMC influences these signaling pathways represents an important direction for future research.

We further showed that PMC could bind to all three isoforms of human KDELR and affect their localization (Figs. 1 and 2, and Supplementary Fig. S1). The specificity of PMC binding to KDELRs was substantiated by the reduction in KDELRs bound to PMC-immobilized beads following addition of free PMC (Fig. 1). Fluorescence imaging also demonstrated that PMC treatment led to KDELR-EGFP distributions outside the cis-Golgi network (Fig. 2). Conventional KDEL ligand binding facilitates the translocation of KDELR to the ER^14,18,46^, whereas KDELR mutants defective in ligand binding or retrograde transport localize within the Golgi^47^. Upon PMC treatment, KDELR-EGFPs showed a punctate distribution near the cis-Golgi as marked by GM130, suggesting that PMC interferes with KDELR trafficking back to the ER. Using the Fluoppi system, we also observed that PMC disrupts the binding of KDELR to the KDEL ligand BiP in a living cell environment (Supplementary Movie S1). This observation provides direct evidence that PMC-induced proteostasis crash is initiated by the physical dissociation of the KDELR-chaperone complex. The pH-dependent conformational changes of KDELRs are important for the KDELR function and cargo retrieval^18,48–50^. Because the capture of KDEL peptides by KDELRs is stabilized in the acidic Golgi lumen, PMC could potentially alter Golgi pH, in addition to directly binding KDELRs, and thereby indirectly affect KDEL ligand binding. Further studies are needed to clarify how PMC binding alters the conformation and trafficking of KDELRs and to investigate the effects of PMC on pH dynamics.

Cancer growth depends on carefully balanced proteostasis because transformation increases the demand for new protein synthesis to support proliferation, migration, and differentiation^51,52^. Mounting evidence indicates that cancer cells are vulnerable to interventions that exacerbate ER stress, leading to terminal UPR^27,32^. For example, inhibition of adaptive UPR signaling and downregulation of ER chaperones increased ER stress and promoted the death of various cancer cel types^35^. In addition, bortezomib, a first-in-class selective proteasome inhibitor, suppresses proteasome-dependent degradation of unfolded proteins and induces terminal UPR to kill cancer cells^27,53,54^. Elevated expression of KDELR isoforms has also been observed in multiple forms of cancer, likely as a compensatory response to enhance retrieval of ER-resident proteins and overcome an imbalanced proteostasis network, thereby maintaining cancer cell survival^8,10,55–62^. Therefore, the pharmacological inhibition of KDELRs to reduce protein-folding capacity in cancer cells is emerging as a novel therapeutic strategy. Our finding that PMC suppresses this proteostatic function of KDELRs supports the idea that drugs targeting KDELR could be novel anticancer agents or chemosensitizers for selectively enhancing ER stress in cancer cells.

Our data shows that PMC enhances XBP1 activation when combined with thapsigargin (Fig. 5c and Supplementary Fig. S2). Given that PMC perturbs KDELR-dependent proteostasis and enhances ER stress, it is important to consider how PMC interacts with other pathways that regulate the cellular stress response. In this context, exploring the combination of PMC with UPR sensor inhibitors, such as IRE1α or PERK inhibitors, represents an intriguing therapeutic strategy. However, as highlighted in recent literature^25,28^, the role of IRE1α in cancer is complex due to its dual capacity for promoting cell survival via XBP1 splicing and inducing apoptosis under persistent stress. Therefore, further studies are necessary to determine whether UPR senser inhibition synergistically amplifies PMC-induced proteostatic stress or interferes with its cell death-inducing pathways.

### Limitations

This study has several limitations. First, the efficacy of KDELR inhibition by PMC as a potential antitumor treatment requires additional confirmation in animal models. Second, the toxicity of PMC at effective doses must be carefully assessed in these models. Third, it is essential to fully evaluate the specificity of PMC binding to KDELR as the nontarget effects of cancer treatments underlie many of the intolerable side effects that limit clinical utility. Moreover, biomarkers predictive of KDELR suppression are required to aid in further studies on PMC, PMC derivatives, and other KDELR inhibitors as anticancer agents.

## Methods

### Cell culture and treatment

The HKe3-mtKRAS cell line was established from HCT116 cells as previously described^11,12^, while wild-type HCT116 and HeLa cells were obtained from the ATCC. All lines were cultured in Dulbecco’s modified Eagle’s medium (Wako Pure Chemical Industries) supplemented with 10% fetal calf serum and penicillin/streptomycin (Gibco) at 37 °C under a humidified 5% CO_2_ atmosphere.

Pyra-metho-carnil (IUPAC Name: 1-{3-[(3,5-dimethylpyrazol-1-yl)methyl]-4-methoxyphenyl}-2,3,4,9-tetrahydro-1H-pyrido[3,4-b]indole) was synthesized by Namiki Shoji^4^ and used at final concentrations of 10, 30, and 60 μM as indicated. The caspase inhibitor z-VAD (OMe)-fmk (Abcam, ab120487) was used at a final concentration of 10 μM and the SERCA inhibitor thapsigargin (T9033; Sigma-Aldrich) at a final concentration of 0.03 μM.

### Constructs

The cDNAs encoding human KDELR1–3 were amplified from the reverse transcription products of Jurkat cells by PCR using the primers shown in Supplementary Table S1, and then cloned into an EGFP-N1 vector (Clontech) between XhoI and EcoRI sites, while cDNAs encoding KDELRs tagged with FLAG at the C-terminus were amplified and cloned into a pcDNA3 vector (Invitrogen) between HindIII and EcoRI sites. All expression vectors were verified by DNA sequencing.

### Antibodies

The following primary antibodies were used in this study: anti-KDELR (Stress Marq, SMC-129), anti-FLAG (M2) (Sigma-Aldrich, F1804), anti-GM130 (BD Biosciences, 610,822), anti-XBP1 (Abcam, ab220783), anti-PERK (Cell Signaling Technologies, #5683), anti-phosphorylated-eIF2alpha (Ser51) (Cell Signaling Technologies, #9721), anti-eIF2alpha (Cell Signaling Technologies, #9722), anti-ATF4 (Cell Signaling Technologies, #11,815), anti-CHOP (Cell Signaling Technologies, #9895), anti-caspase-3 (Cell Signaling Technologies, #9665), anti-cleaved caspase-3 (Cell Signaling Technologies, #9664), anti-PARP (Cell Signaling Technologies, #9542), and anti-actin (Sigma-Aldrich, A2066). The following secondary antibodies were also used: horseradish peroxidase (HRP)-conjugated goat anti-mouse IgG (Jackson, 115-035-003), HRP-conjugated goat anti-rabbit IgG (Jackson ImmunoResearch, 111-035-003), and Alexa555-conjugated goat anti-mouse IgG (Molecular Probes, A21424).

### Pull-down and MS analysis

Pyra-metho-carnil was conjugated to magnetic beads (FG linker beads, Tamagawa Seiki, TAS8848N1110) by mixing in immobilization buffer [10 mM HEPES–NaOH (pH 7.9)]. Cells were lysed in binding/washing buffer [20 mM HEPES–NaOH (pH 7.9), 150 mM KCl, 1 mM MgCl_2_, 0.2 mM CaCl_2_, 0.2 mM EDTA, 10% glycerol, and 0.1% NP-40] supplemented with cOmplete EDTA-Free Protease Inhibitor (Roche, 05 056 489 001) by two, 30-s periods of sonication using a Bioruptor (Cosmo Bio), followed by incubation for 30 min at 4 °C. The cell pellet was then removed by centrifugation and the soluble fraction separated into two samples. One sample was directly incubated with PMC-conjugated FG linker beads for 2 h at 4 °C while the other was first mixed with the indicated concentrations of free PMC as a comparator. Both samples were then separated by SDS-PAGE under the same conditions. Briefly, the beads were washed three times with binding/washing buffer and boiled in Laemmli sample buffer to elute PMC-binding proteins. The eluates were separated by SDS-PAGE, followed by silver staining or immunoblotting. A full-length gel image and uncropped images of the trimmed blots are provided in Supplementary Information Fig. 1b and d. The silver-stained gel was used for MS sample preparation. Gel slices containing individual protein bands from untreated lysate but absent in samples first mixed with free PMC (Fig. 1b, arrow) were excised and the proteins subjected to trypsin digestion and nano liquid chromatography-tandem mass spectrometry (nano-LC/MS/MS) analysis by standard protocol (Japan Proteomics Co. Ltd, Sendai).

### DNA and siRNA transfections

To express FLAG-tagged KDELR1–3 (Fig. 1e), cells were transfected with the indicated expression vector using polyethyleneimine “MAX” transfection reagent (Polysciences) as previously described^63^. For immunofluorescence analysis of EGFP-tagged KDELR1–3 (Fig. 2a, b), cells were transfected for 6 h using Lipofectamine 3000 (Invitrogen) as previously described^64^, followed by incubation in fresh medium. For suppression of CHOP and KDELR expression, cells were transfected with targeted small interfering (si) RNAs obtained from Thermo Scientific and Dharmacon, respectively. The siRNA sequences are shown in Supplementary Table S2. For KDELR knockdown, cells were transfected with the following siRNA mixtures (siKDELR mixture #1: KDELR1 #3 + KDELR2 #1 + KDELR3 #1; siKDELR mixture #2: KDELR1 #4 + KDELR2 #2 + KDELR3 #3) using Lipofectamine RNAiMAX (Invitrogen) according to the manufacturer’s reverse protocol. To confirm siRNA-induced knockdown of CHOP protein expression (Fig. 4c), cells were seeded in six-well plates at 5 × 10^5^ cells/well in 2.5 mL culture medium with siRNA (10 pmol)–Lipofectamine RNAiMAX (5 µL) complexes, followed by immunoblotting as described in the next section. To examine the effects of CHOP knockdown on cell death (Fig. 4d), cells were seeded in 24-well plates at 5 × 10^4^ cells/well in 0.5 mL of culture medium with siRNA (2 pmol)–Lipofectamine RNAiMAX (1 µL) complexes. After 24 h, the culture medium was replaced with fresh medium containing 60 µM PMC, and the cells were incubated for an additional 24 h, followed by viable cell counting assays as described in subsequent sections. To confirm siRNA-mediated knockdown of KDELR protein expression levels (Fig. 6a), cells were seeded in six-well plates at 5 × 10^5^ cells/well in 2.5 mL culture medium containing siRNA (10 pmol for each isoform, total 30 pmol)–Lipofectamine RNAiMAX (5 µL) complexes. Cells were harvested 48 h after transfection and analyzed by immunoblotting.

### Immunoblotting

Cell lysates were prepared and subjected to immunoblotting as previously described^64^. For the experiments shown in Figs. 3, 4, 5, cells treated as indicated were lysed in RIPA buffer (50 mM Tris–HCl, pH 7.5, 150 mM NaCl, 0.5% sodium deoxycholate, 0.1% SDS, and 1% Triton X-100) supplemented with cOmplete EDTA-free protease inhibitors. In Figs. 1 and 6, cells were lysed in binding/washing buffer as described in the pull-down assay section. The membranes were trimmed prior to antibody incubation in order to probe different molecular weight regions separately. Consequently, full-length membrane images are not available for these blots. Uncropped images of the trimmed membrane pieces, as well as all repeat experiments, are provided in the Supplementary Information.

### Immunofluorescence analysis and protein aggregation detection assay

Immunofluorescence analysis was performed as previously described with minor modifications^65,66^. Cells were seeded on 12-mm diameter glass coverslips placed in 24-well plates for 48 h. After a brief wash with phosphate-buffered saline (PBS), the cells were fixed with 4% paraformaldehyde in PBS for 15 min at room temperature (RT), washed three times with PBS, permeabilized with permeabilization/blocking buffer (1% nonfat dry milk in PBS containing 0.3% Triton X-100) for 30 min at RT, and subsequently incubated with an anti-GM130 antibody diluted in permeabilization/blocking buffer for 1 h at RT. Following incubation, the cells were washed thrice with PBS and subsequently incubated with a fluorophore-conjugated secondary antibody in permeabilization/blocking buffer for 1 h at RT, washed three times with PBS, counterstained with 4’,6-diamidino-2-phenylindole (DAPI), mounted using Fluorescence Mounting Medium (Dako, S3023), and examined using a TCS SP5 laser-scanning confocal microscope with a 63 × /1.4 NA oil objective lens (Leica Microsystems).

Protein aggregates were detected using the PROTEOSTAT Aggresome Detection kit (Enzo Life Sciences, ENZ-51035-0025) according to the manufacturer’s instructions. Briefly, cells were seeded on glass coverslips for 30 h, washed with PBS, fixed with 4% paraformaldehyde in PBS for 30 min at RT, permeabilized with PBS containing 0.5% Triton X-100 and 3 mM EDTA for 30 min on ice, stained with the PROTEOSTAT dye for 30 min at RT, counterstained with DAPI, mounted, and analyzed as described for KDELRs. The fluorescence emission intensities of PROTEOSTAT were analyzed using ImageJ software (National Institute of Health).

### Fluoppi assay for detection of protein–protein interactions

To analyze the interaction between KDELR1 and BiP using the Fluoppi (fluorescence-based technology detecting protein–protein interactions) system, expression vectors encoding KDELR1 fused to the homomeric assembly tag (hAG) and BiP fused to the assembly helper tag (Ash) were constructed as follows. The cDNA corresponding to the full-length of human KDELR1 was amplified by PCR using HCT116 cell-derived cDNA as a template with the primers Bam-KDELR1 Fw (5′-ATAAGGATCCGCCATGAATCTCTTCCGATTCCTGGGAG-3′) and KDELR1-XhoI Rv (5′-GGGAAGAAGTTGAGTTTGCCGGCAggCTCGAGATTA-3′). The amplified fragment was inserted into the BamHI/XhoI sites of the phAG-MNLinker vector to generate KDELR1-hAG. Similarly, the cDNA corresponding to the full-length of human BiP was amplified from HCT116 cell cDNA using the primers Bam-BiP Fw (5′-ATAAggatccATGAAGCTCTCCCTGGTGGCCGCGAT-3′) and BiP-XhoI Rv (5′-TACAGCAGAAAAAGATGAGTTGTAGctcgagaTTA-3′), and the amplified fragment was cloned into the BamHI/XhoI sites of the pAsh-MCLinker vector to produce Ash-BiP.

HCT116 cells were transfected with KDELR1-hAG alone or co-transfected with KDELR1-hAG and Ash-BiP expression plasmids using Lipofectamine 3000. After 24 h, transfected cells were subjected to selection with 2 μg/mL puromycin for 2 weeks, and the resulting stable cell lines were designated as HCT116K and HCT116KB, respectively. HCT116K and HCT116KB cells were seeded onto 8-well chamber slides and cultured overnight. Cells were then treated with 30 µM PMC, and time-lapse imaging of Fluoppi puncta was performed using a BZ-X700 fluorescence microscope (Keyence). Fluorescence and bright-field images were captured every 1 min, and time-lapse movies were subsequently generated.

### Trypan-blue exclusion assay

For viable cell counting, cells treated as indicated were detached from the culture substrate by trypsinization and the cell suspension mixed with an equal volume of 0.4% trypan blue solution (Wako Pure Chemical Industries, 207-17081). Viable (unstained) and nonviable (stained) cells were counted using a hemocytometer and the percentage of nonviable cells was calculated.

### CCK-8 assay

Cell proliferation rate was measured using the Cell Counting Kit-8 (CCK-8, Dojindo) according to the manufacturer’s protocol. Briefly, to examine the effects of PMC and thapsigargin on cell proliferation (Fig. 5), cells were seeded in 96-well plates at 5 × 10^3^ cells per well in 100 μL culture medium for 24 h. The plating medium was then replaced with fresh medium containing vehicle, PMC, or thapsigargin as indicated and cells incubated for 48 h, followed by CCK-8 assays. The GI_50_ values were calculated from the linear portion of the dose–response curve. To examine the effect of KDELR siRNAs on cell proliferation (Fig. 6b), cells were seeded in 96-well plates at 1 × 10^4^ cells/well in 100 μL culture medium with siRNA (0.4 pmol for each isoform, total 1.2 pmol)–Lipofectamine RNAiMAX (0.2 μL) for 72 h, followed by CCK-8 assays as described above.

### Statistical analysis

All datasets are presented as mean ± standard deviation (SD). Treatment groups were compared by two-tailed independent samples Student’s t-test with statistical significance set at *p* < 0.05.

## Supplementary Information

Below is the link to the electronic supplementary material.

Supplementary Material 1

Supplementary Material 2

Supplementary Material 3

Supplementary Material 4

## Data Availability

All data supporting the conclusions of this work are included in the main manuscript or online supplementary information. Further inquiries can be directed to the corresponding authors.
