# Supplementary material for: Pyra-metho-carnil disrupts cancer cell proteostasis and induces apoptosis by binding to KDEL receptors
Source: Sci Rep. 2026 Mar 26;16:15145. doi: 10.1038/s41598-026-45604-z (PMC13172550; doi:10.1038/s41598-026-45604-z)
Supplement: Supplementary file 1 — Supplementary Material 1 [file 41598_2026_45604_MOESM1_ESM.docx]

**Supplementary Table S1. Primers**

| **Name** | **Sequence (5’ to 3’)** |
| --- | --- |
| KDELR1-N (XhoI/EGFP) | TTTCTCGAGGCCGCCACCATGAATCTCTTCCGATTCCTGGG |
| KDELR1-C2 (EcoRI/EGFP) | AAAGAATTCCTGCCGGCAAACTCAACTTCTTCCC |
| KDELR1-N2 (HindIII/pcDNA3) | TTTAAGCTTGCCGCCACCATGAATCTCTTCCGATTCCTGGG |
| KDELR1-C1 (EcoRI/pcDNA3) | TTTGAATTCTCACTTGTCGTCATCGTCTTTGTAGTCTGCCGGCAAACTCAACTTCTTCCC |
| KDELR2-N (XhoI/EGFP) | TTTCTCGAGGCCGCCACCATGAACATTTTCCGGCTGACTGGG |
| KDELR2-C2 (EcoRI/EGFP) | AAAGAATTCCTGCTGGCAAACTGAGCTTCTTTCCC |
| KDELR2-N2 (HindIII/pcDNA3) | TTTAAGCTTGCCGCCACCATGAACATTTTCCGGCTGACTGGG |
| KDELR2-C1 (EcoRI/pcDNA3) | TTTGAATTCTCACTTGTCGTCATCGTCTTTGTAGTCTGCTGGCAAACTGAGCTTCTTTCCC |
| KDELR3-N (XhoI/EGFP) | TTTCTCGAGGCCGCCACCATGAACGTGTTCCGAATCCTCGGCG |
| KDELR3-1-C2 (EcoRI/EGFP) | AAAGAATTCCGATTGGCATTGGAAGACTTAACTTC |
| KDELR3-N2 (HindIII/pcDNA3) | TTTAAGCTTGCCGCCACCATGAACGTGTTCCGAATCCTCGGCG |
| KDELR3-1-C1 (EcoRI/pcDNA3) | TTTGAATTCTCACTTGTCGTCATCGTCTTTGTAGTCGATTGGCATTGGAAGACTTAACTTC |
