# Supplementary material for: Pyra-metho-carnil disrupts cancer cell proteostasis and induces apoptosis by binding to KDEL receptors
Source: Sci Rep. 2026 Mar 26;16:15145. doi: 10.1038/s41598-026-45604-z (PMC13172550; doi:10.1038/s41598-026-45604-z)
Supplement: Supplementary file 2 — Supplementary Material 2 [file 41598_2026_45604_MOESM2_ESM.docx]

| **Name** | **Company** | **Catalog #** | **Sequence (5’ to 3’)** |
| --- | --- | --- | --- |
| siCHOP #1 | Thermo Scientific | Stealth RNAi siRNA, DDIT3 VHS40605 | GAGAAUGAACGGCUCAAGCAGGAAA |
| siCHOP #2 | Thermo Scientific | Stealth RNAi siRNA, DDIT3 VHS40607 | CCAGGAAACGGAAACAGAGUGGUCA |
| siKDELR1 #3 | Dharmacon | ON-TARGETplus, J-019136-10 | AGUUGAGUUUGCCGGCAUA |
| siKDELR1 #4 | Dharmacon | ON-TARGETplus, J-019136-12 | CCACGGUCUGGUUGAUUUA |
| siKDELR2 #1 | Dharmacon | ON-TARGETplus, J-012315-05 | GCACUGGUCUUCACAACUC |
| siKDELR2 #2 | Dharmacon | ON-TARGETplus, J-012315-07 | UACCUGAAAUUUAAGGCAA |
| siKDELR3 #1 | Dharmacon | ON-TARGETplus, J-012316-05 | CAGUGUACAUGAUAUAUGG |
| siKDELR3 #3 | Dharmacon | ON-TARGETplus, J-012316-07 | GGUACCAGACUGAGAAUUU |
| siControl | Dharmacon | ON-TARGETplus Non-targeting Pool, D-001810-10-05 | UGGUUUACAUGUCGACUAA, UGGUUUACAUGUUGUGUGA, UGGUUUACAUGUUUUCUGA, UGGUUUACAUGUUUUCCUA |

**Supplemental Table S2. siRNAs**
