# Supplementary material for: Pyra-metho-carnil disrupts cancer cell proteostasis and induces apoptosis by binding to KDEL receptors
Source: Sci Rep. 2026 Mar 26;16:15145. doi: 10.1038/s41598-026-45604-z (PMC13172550; doi:10.1038/s41598-026-45604-z)
Supplement: Supplementary file 3 — Supplementary Material 3 [file 41598_2026_45604_MOESM3_ESM.pptx]

## Slide 1
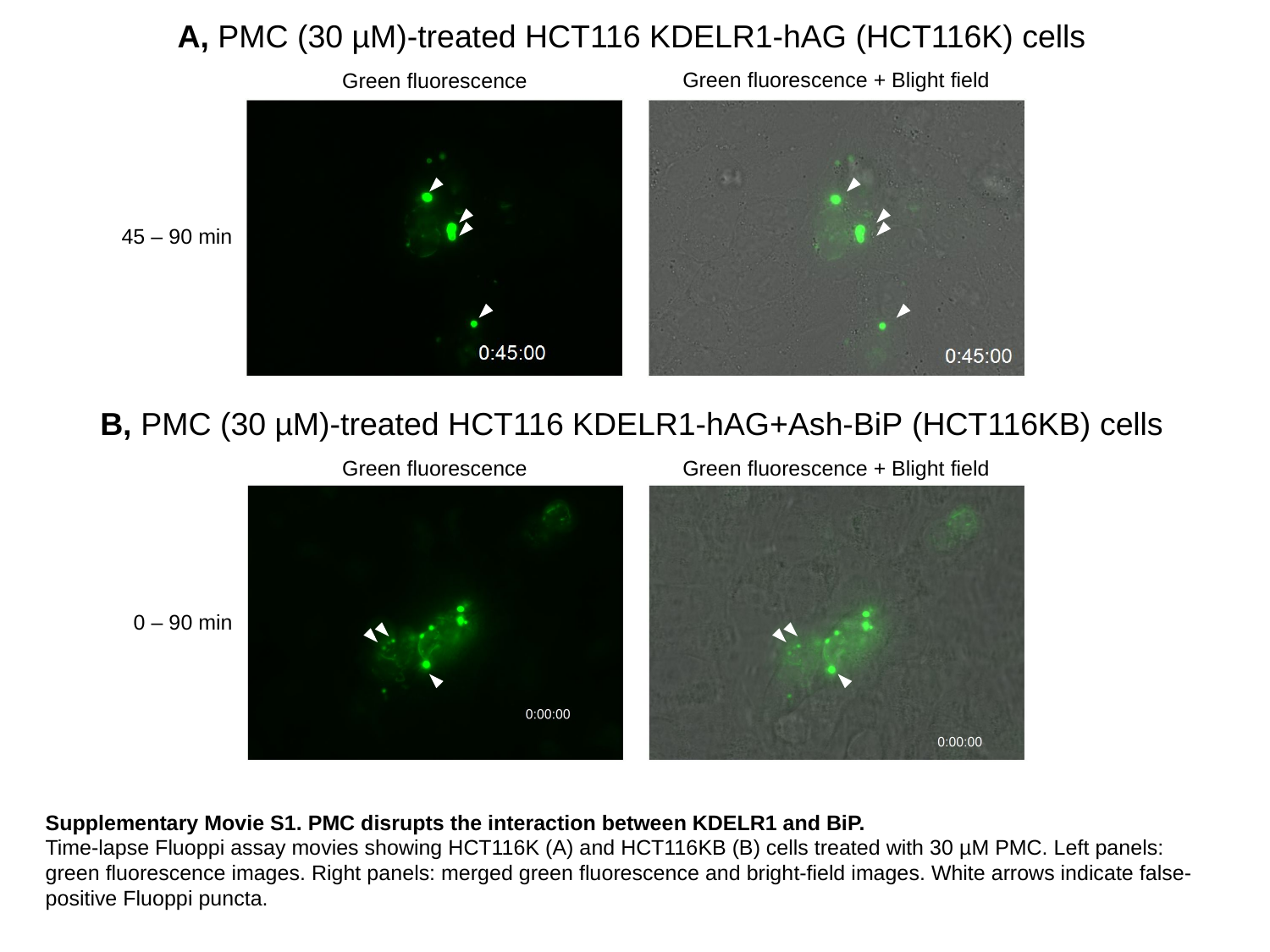

A, PMC (30 µM)-treated HCT116 KDELR1-hAG (HCT116K) cells
Green fluorescence + Blight field
Green fluorescence
45 – 90 min
B, PMC (30 µM)-treated HCT116 KDELR1-hAG+Ash-BiP (HCT116KB) cells
Green fluorescence
Green fluorescence + Blight field
0 – 90 min
Supplementary Movie S1. PMC disrupts the interaction between KDELR1 and BiP.
Time-lapse Fluoppi assay movies showing HCT116K (A) and HCT116KB (B) cells treated with 30 µM PMC. Left panels: green fluorescence images. Right panels: merged green fluorescence and bright-field images. White arrows indicate false-positive Fluoppi puncta.
