# Supplementary material for: Pyra-metho-carnil disrupts cancer cell proteostasis and induces apoptosis by binding to KDEL receptors
Source: Sci Rep. 2026 Mar 26;16:15145. doi: 10.1038/s41598-026-45604-z (PMC13172550; doi:10.1038/s41598-026-45604-z)
Supplement: Supplementary file 4 — Supplementary Material 4 [file 41598_2026_45604_MOESM4_ESM.pptx]

## Slide 1
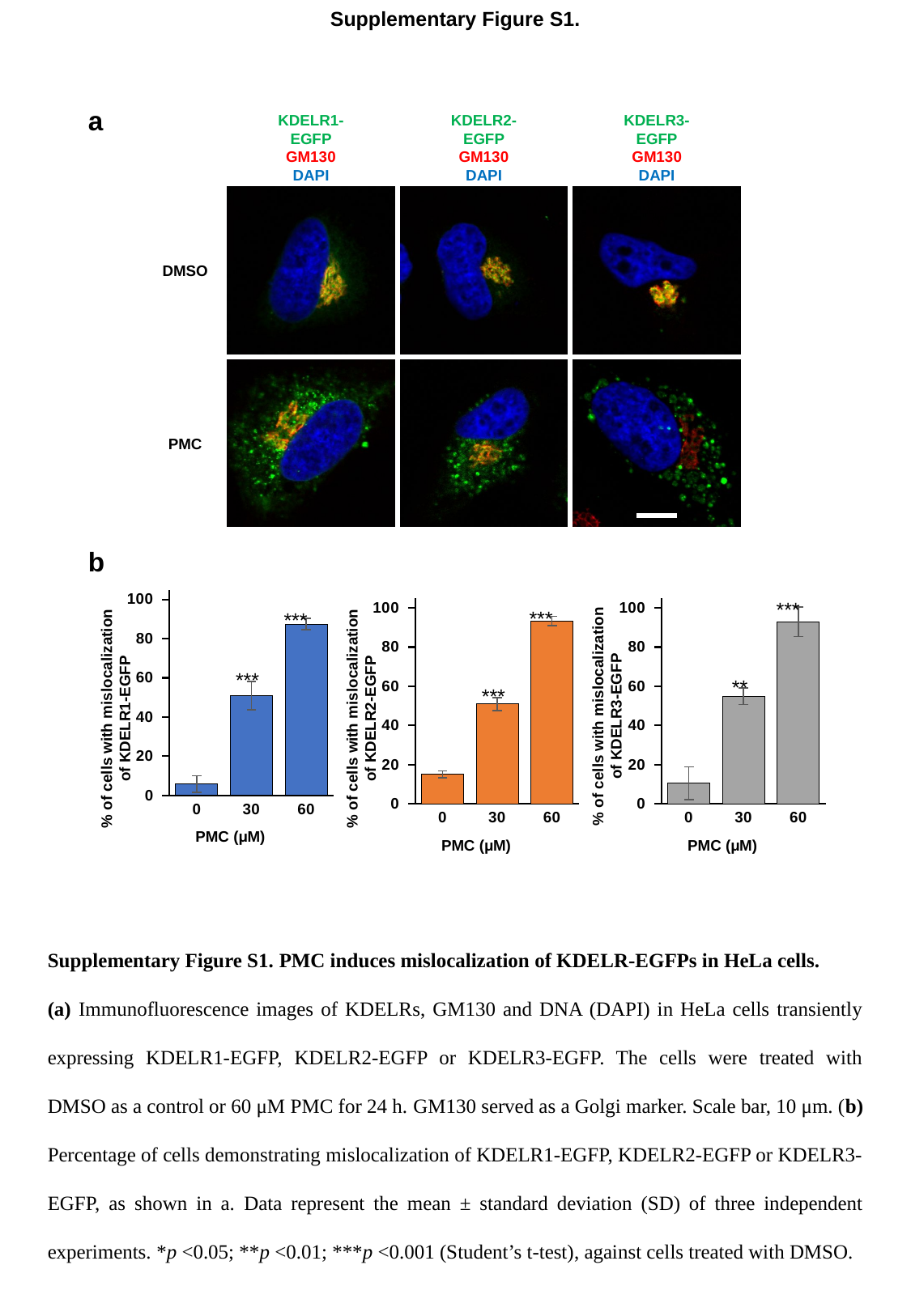

Supplementary Figure S1.
a
KDELR1-EGFP
GM130
DAPI
KDELR2-EGFP
GM130
DAPI
KDELR3-EGFP
GM130
DAPI
DMSO
PMC
b
### Chart
| Category | KDELR1-EGFP |
|---|---|
| 0 | 5.849999999999999 |
| 30 | 50.89938271604939 |
| 60 | 87.40333333333332 |***
### Chart
| Category | KDELR2-EGFP |
|---|---|
| 0 | 15.044496124031008 |
| 30 | 50.99666666666667 |
| 60 | 93.25888888888888 |
### Chart
| Category | KDELR3-EGFP |
|---|---|
| 0 | 10.605555555555556 |
| 30 | 54.86666666666667 |
| 60 | 92.91666666666667 |***
***
***
**
***
% of cells with mislocalization of KDELR3-EGFP
% of cells with mislocalization of KDELR2-EGFP
% of cells with mislocalization of KDELR1-EGFP
Supplementary Figure S1. PMC induces mislocalization of KDELR-EGFPs in HeLa cells.
(a) Immunofluorescence images of KDELRs, GM130 and DNA (DAPI) in HeLa cells transiently expressing KDELR1-EGFP, KDELR2-EGFP or KDELR3-EGFP. The cells were treated with DMSO as a control or 60 μM PMC for 24 h. GM130 served as a Golgi marker. Scale bar, 10 μm. (b) Percentage of cells demonstrating mislocalization of KDELR1-EGFP, KDELR2-EGFP or KDELR3-EGFP, as shown in a. Data represent the mean ± standard deviation (SD) of three independent experiments. *p <0.05; **p <0.01; ***p <0.001 (Student’s t-test), against cells treated with DMSO.

## Slide 2
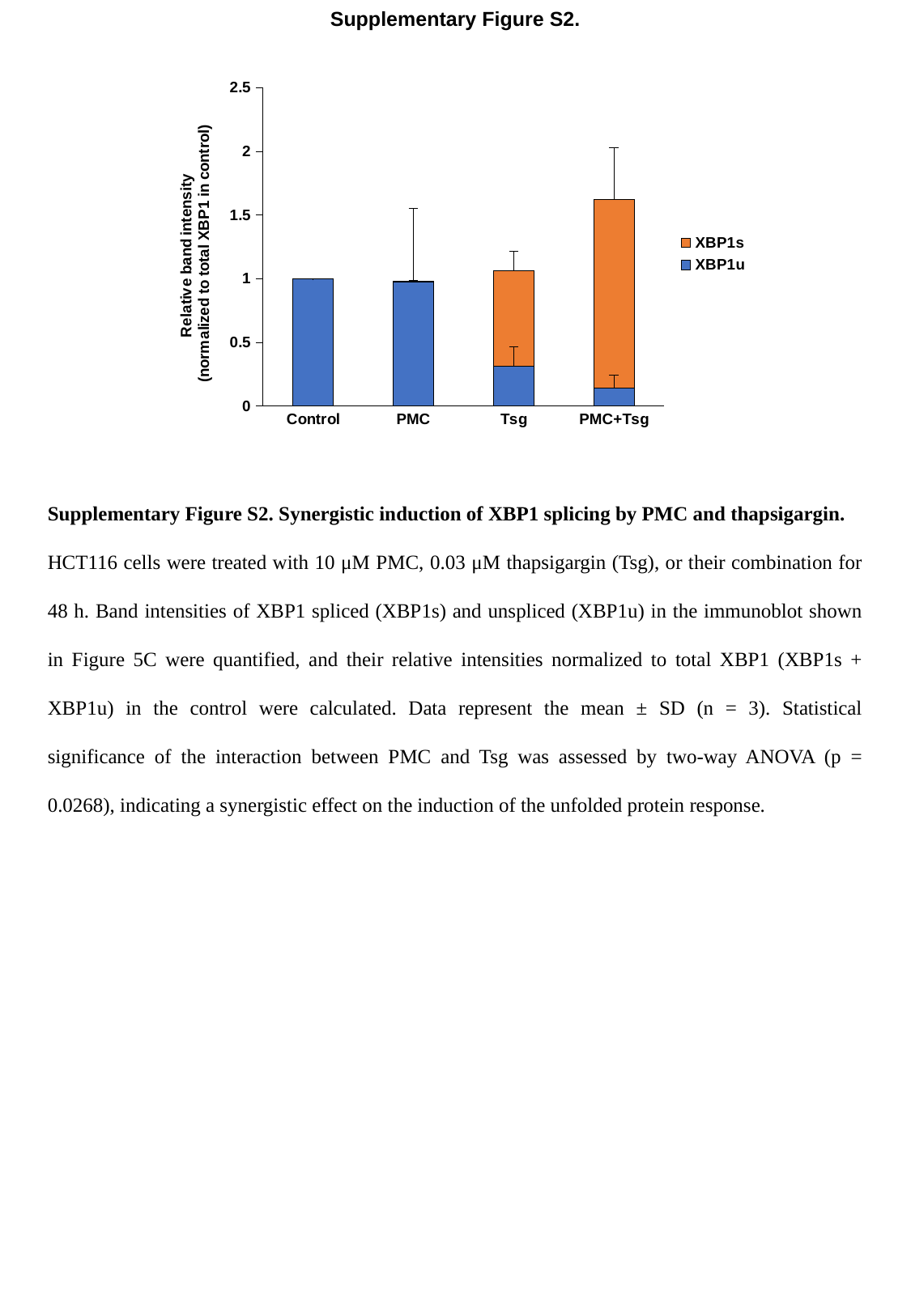

Supplementary Figure S2.
### Chart
| Category | XBP1u | XBP1s |
|---|---|---|
| Control | 0.9967262853250398 | 0.0032737146749600987 |
| PMC | 0.9769603531928016 | 0.004726704692176551 |
| Tsg | 0.31085674939834557 | 0.7554455683484821 |
| PMC+Tsg | 0.14319550755273427 | 1.478543050092785 |Supplementary Figure S2. Synergistic induction of XBP1 splicing by PMC and thapsigargin.
HCT116 cells were treated with 10 μM PMC, 0.03 μM thapsigargin (Tsg), or their combination for 48 h. Band intensities of XBP1 spliced (XBP1s) and unspliced (XBP1u) in the immunoblot shown in Figure 5C were quantified, and their relative intensities normalized to total XBP1 (XBP1s + XBP1u) in the control were calculated. Data represent the mean ± SD (n = 3). Statistical significance of the interaction between PMC and Tsg was assessed by two-way ANOVA (p = 0.0268), indicating a synergistic effect on the induction of the unfolded protein response.

## Slide 3
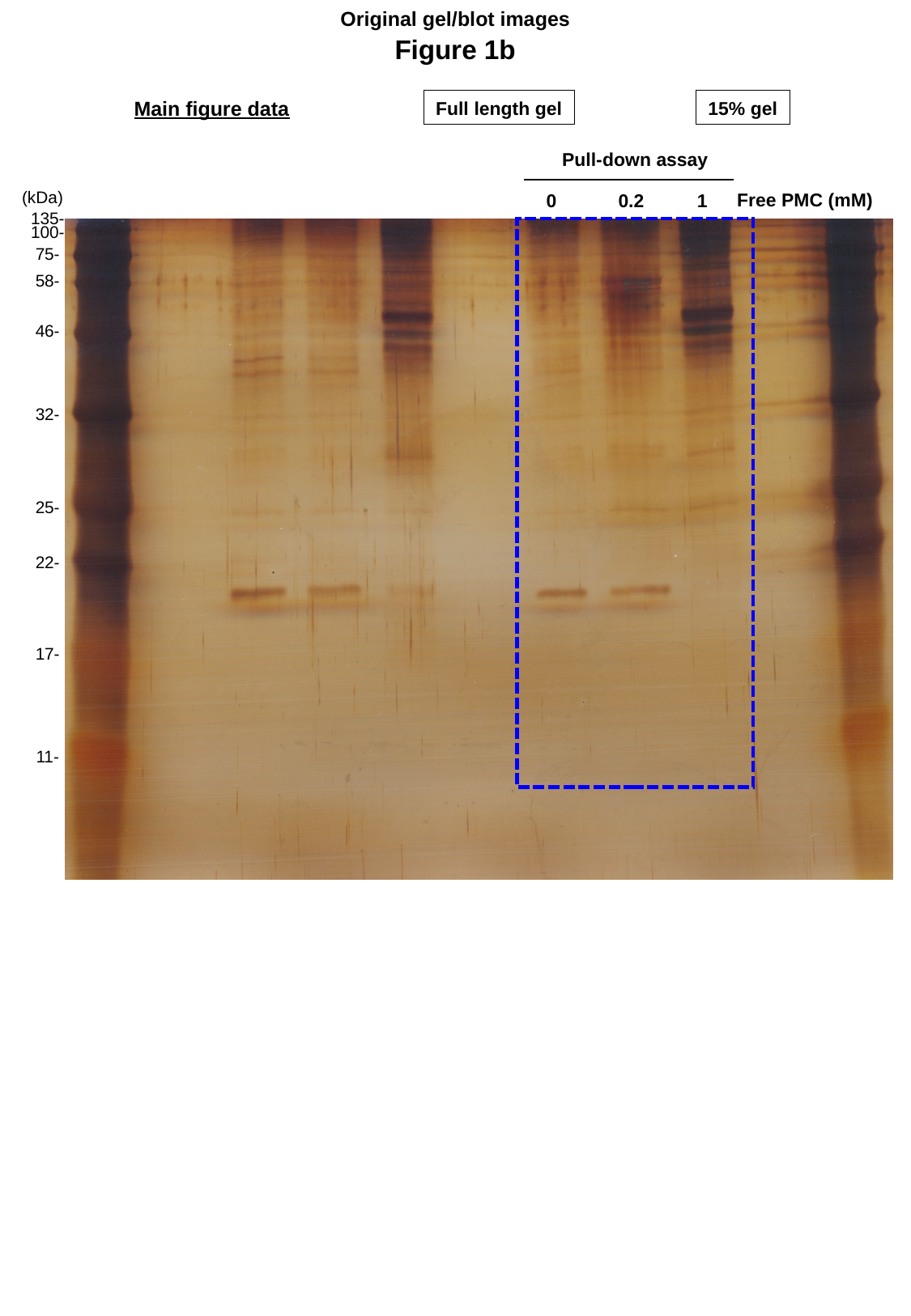

Original gel/blot images
Figure 1b
Main figure data
Full length gel
15% gel
Pull-down assay
(kDa)
Free PMC (mM)
0
0.2
1
135-
100-
75-
58-
46-
32-
25-
22-
17-
11-

## Slide 4
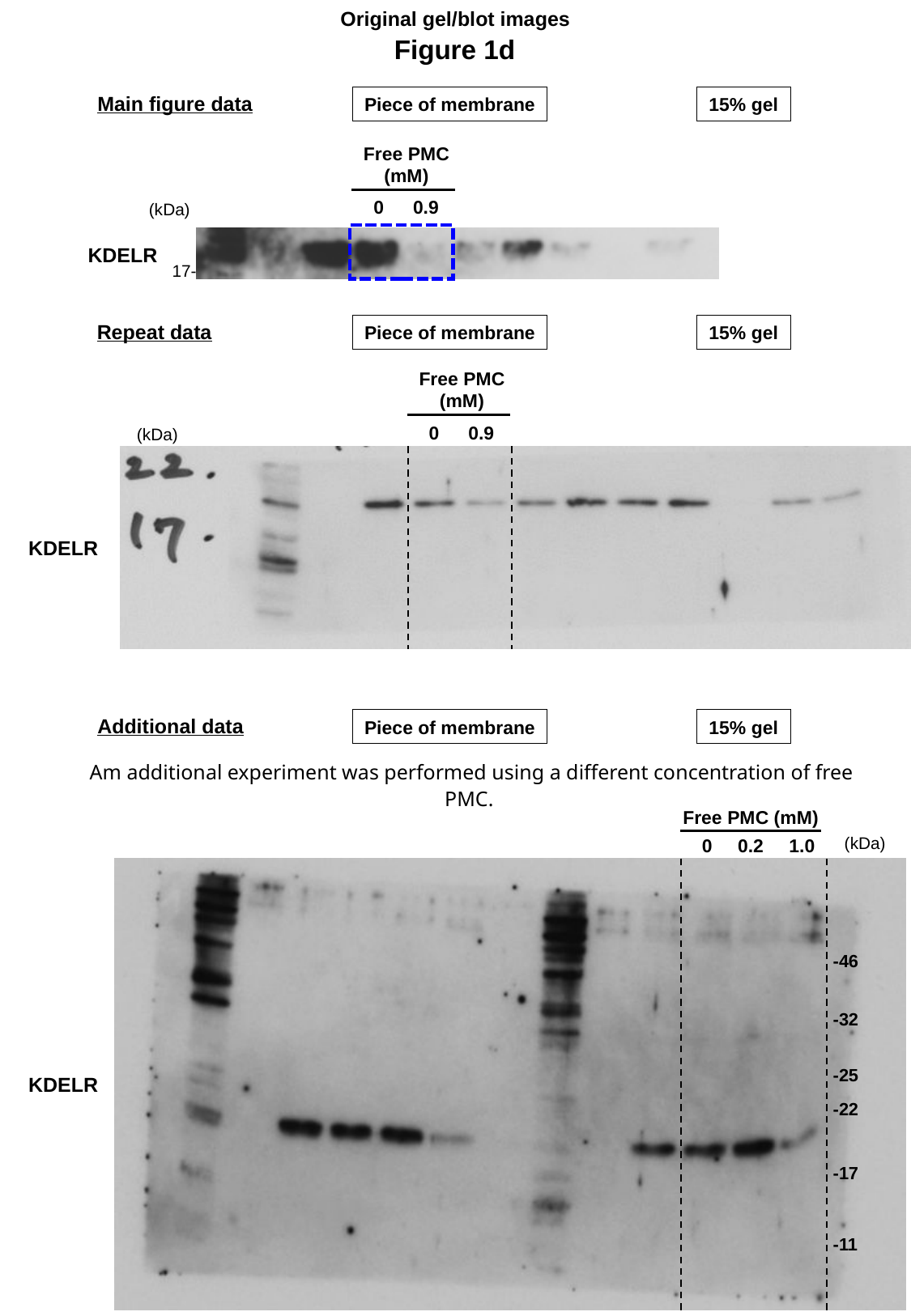

Original gel/blot images
Figure 1d
Main figure data
Piece of membrane
15% gel
Free PMC
(mM)
0
0.9
(kDa)
17-
KDELR
Repeat data
Piece of membrane
15% gel
Free PMC
(mM)
0
0.9
(kDa)
KDELR
Additional data
Piece of membrane
15% gel
Am additional experiment was performed using a different concentration of free PMC.
Free PMC (mM)
(kDa)
0
0.2
1.0
-46
-32
-25
KDELR
-22
-17
-11

## Slide 5
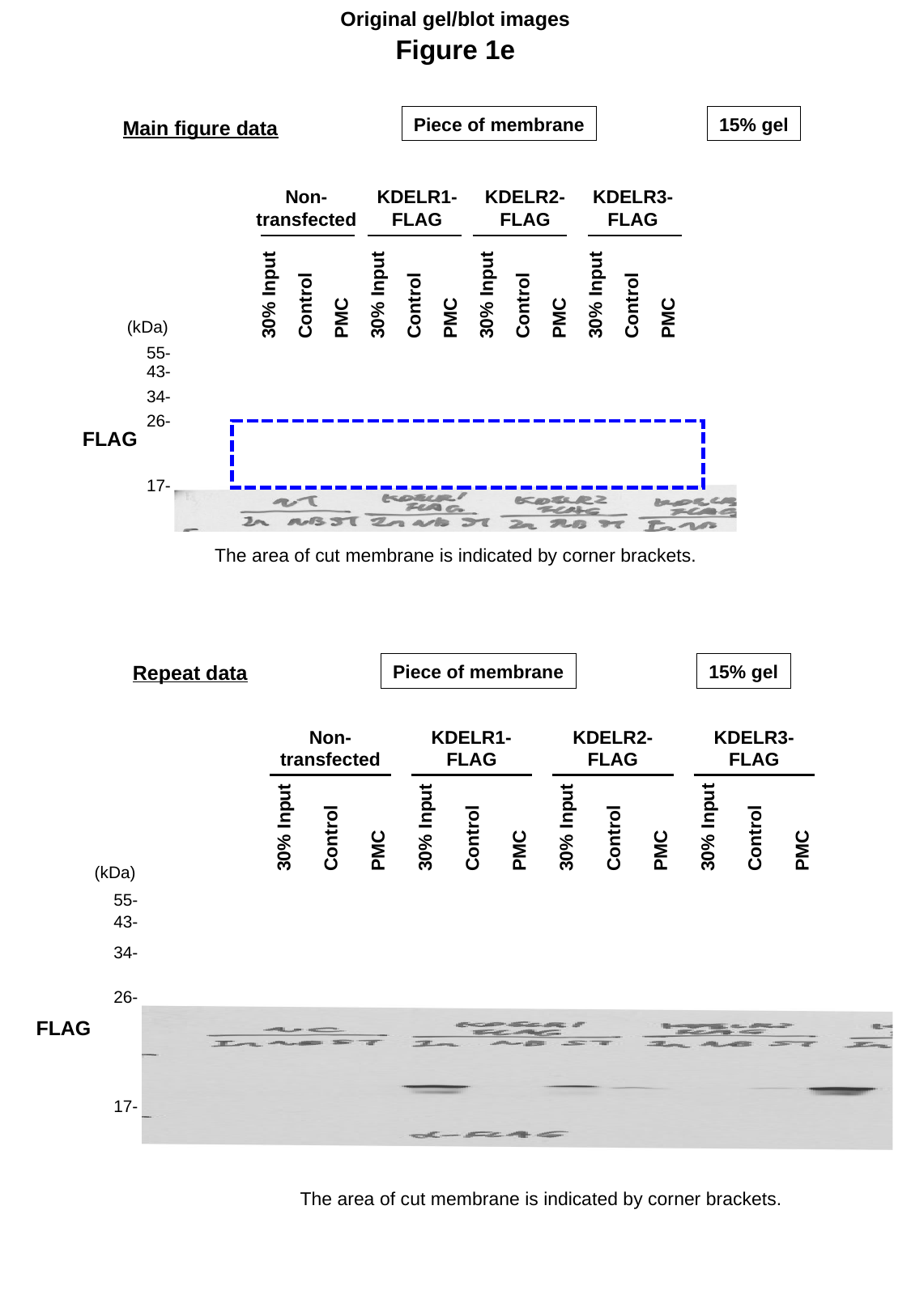

Original gel/blot images
Figure 1e
Piece of membrane
15% gel
Main figure data
Non-transfected
KDELR1-
FLAG
KDELR2-
FLAG
KDELR3-
FLAG
30% Input
30% Input
30% Input
30% Input
Control
Control
Control
Control
PMC
PMC
PMC
PMC
(kDa)
55-
43-
34-
26-
FLAG
17-
The area of cut membrane is indicated by corner brackets.
Repeat data
Piece of membrane
15% gel
Non-transfected
KDELR1-
FLAG
KDELR2-
FLAG
KDELR3-
FLAG
30% Input
30% Input
30% Input
30% Input
Control
Control
Control
Control
PMC
PMC
PMC
PMC
(kDa)
55-
43-
34-
26-
FLAG
17-
The area of cut membrane is indicated by corner brackets.

## Slide 6
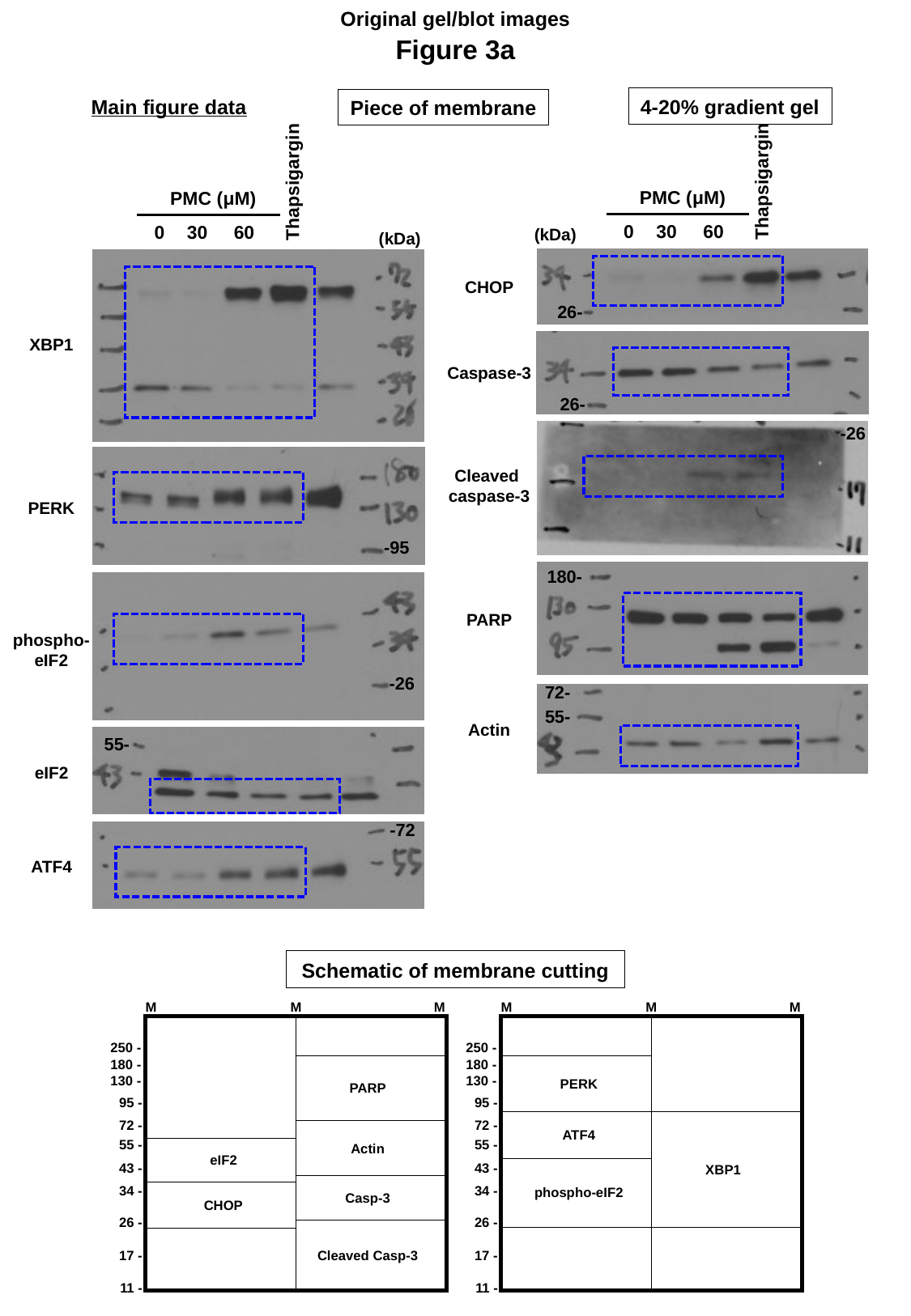

Original gel/blot images
Figure 3a
Main figure data
4-20% gradient gel
Piece of membrane
Thapsigargin
Thapsigargin
PMC (μM)
PMC (μM)
0
30
60
0
30
60
(kDa)
(kDa)
26-
CHOP
XBP1
26-
Caspase-3
-26
-95
Cleaved
caspase-3
PERK
180-
-26
PARP
phospho-
eIF2
72-
55-
Actin
55-
eIF2
-72
ATF4
Schematic of membrane cutting
M
M
M
M
M
M
250 -
250 -
180 -
180 -
130 -
130 -
PERK
PARP
95 -
95 -
72 -
72 -
ATF4
55 -
55 -
Actin
eIF2
43 -
43 -
XBP1
34 -
34 -
phospho-eIF2
Casp-3
CHOP
26 -
26 -
17 -
Cleaved Casp-3
17 -
11 -
11 -

## Slide 7
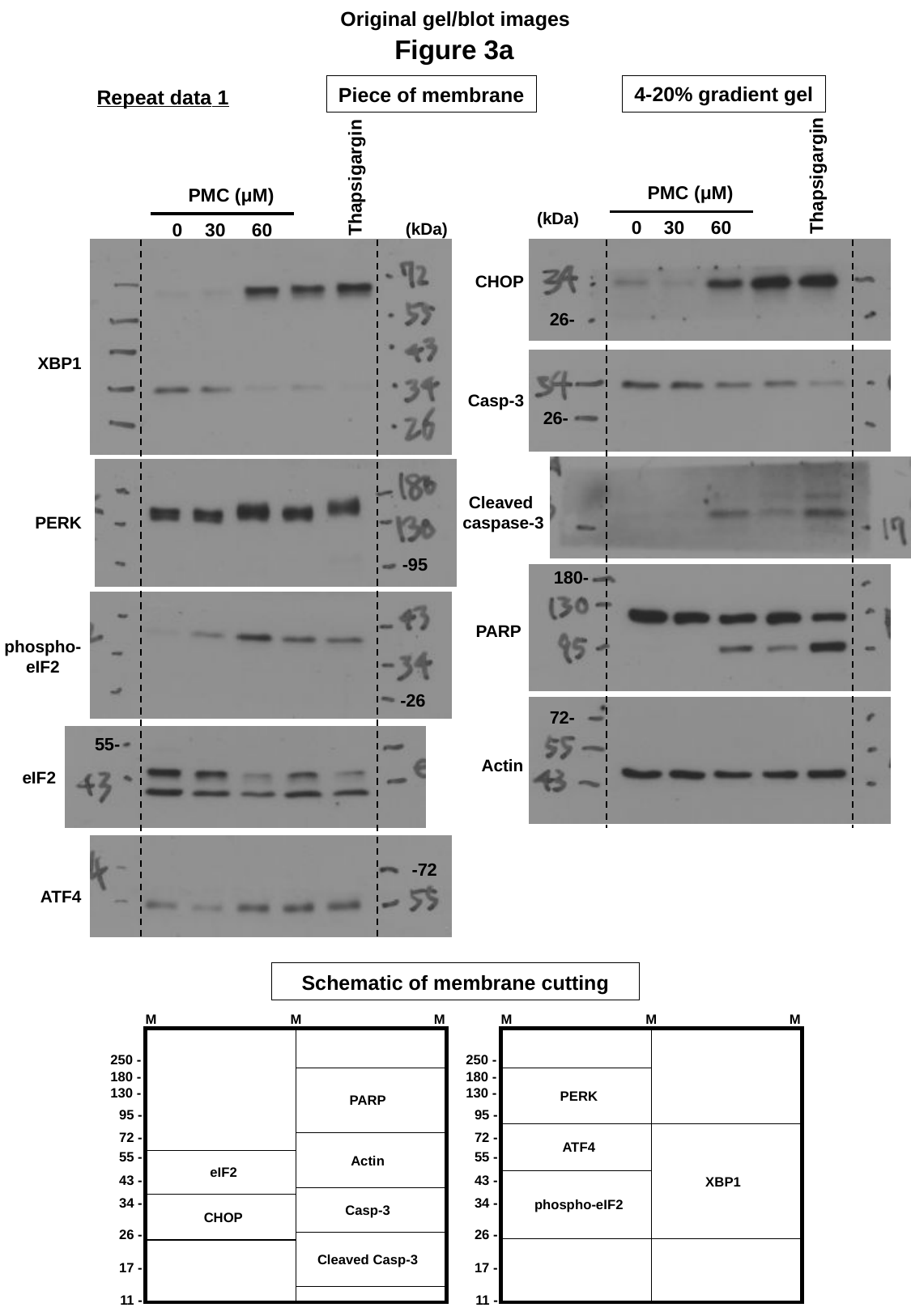

Original gel/blot images
Figure 3a
4-20% gradient gel
Piece of membrane
Repeat data 1
Thapsigargin
Thapsigargin
PMC (μM)
PMC (μM)
(kDa)
0
30
60
(kDa)
0
30
60
CHOP
26-
XBP1
Casp-3
26-
Cleaved
caspase-3
PERK
-95
180-
PARP
phospho-
eIF2
-26
72-
55-
Actin
eIF2
-72
ATF4
Schematic of membrane cutting
M
M
M
M
M
M
250 -
250 -
180 -
180 -
130 -
130 -
PERK
PARP
95 -
95 -
72 -
72 -
ATF4
55 -
55 -
Actin
eIF2
43 -
43 -
XBP1
34 -
34 -
phospho-eIF2
Casp-3
CHOP
26 -
26 -
Cleaved Casp-3
17 -
17 -
11 -
11 -

## Slide 8
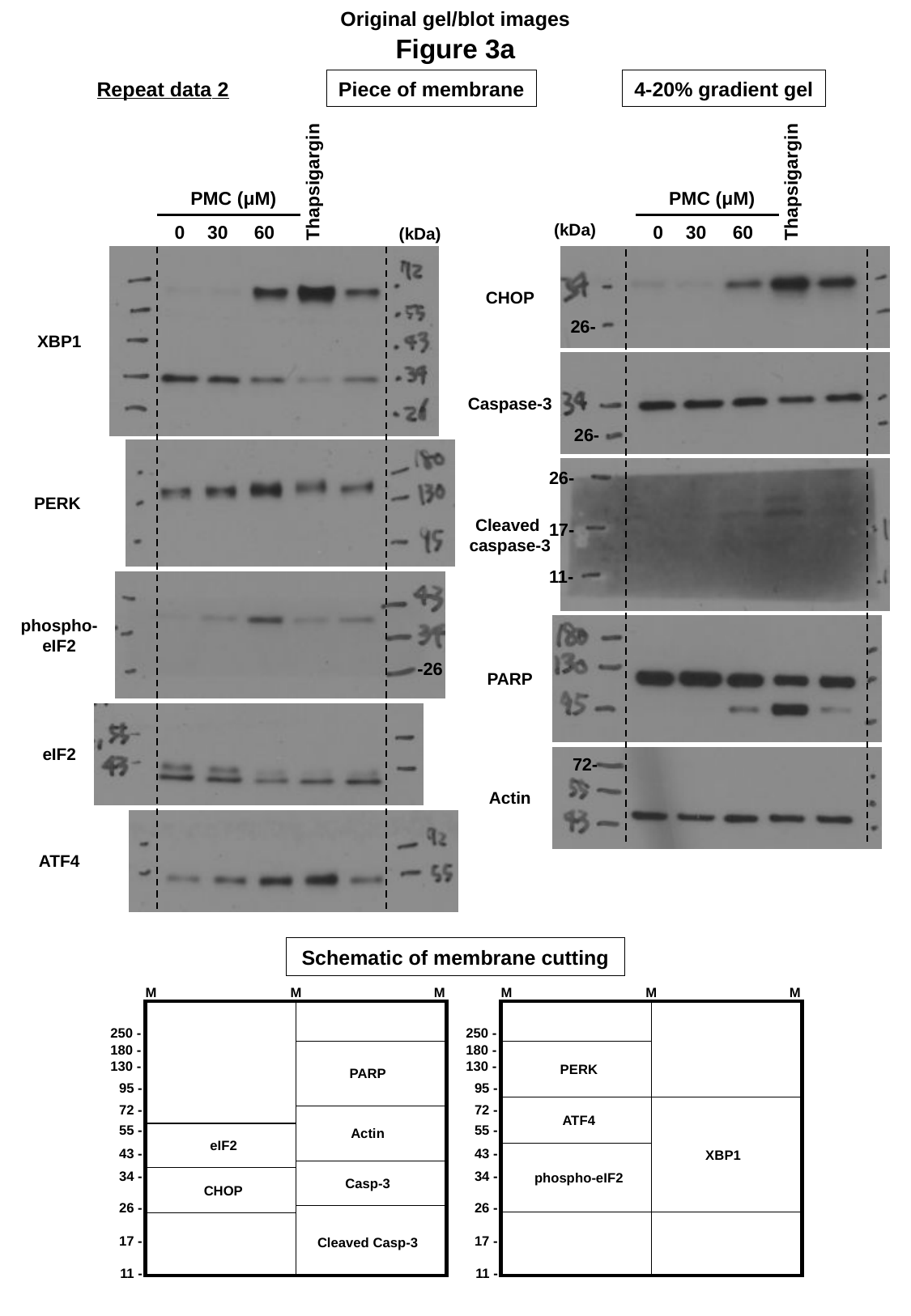

Original gel/blot images
Figure 3a
Repeat data 2
Piece of membrane
4-20% gradient gel
Thapsigargin
Thapsigargin
PMC (μM)
PMC (μM)
(kDa)
0
30
60
0
30
60
(kDa)
CHOP
26-
XBP1
Caspase-3
26-
26-
PERK
Cleaved
caspase-3
17-
11-
phospho-
eIF2
-26
PARP
eIF2
72-
Actin
ATF4
Schematic of membrane cutting
M
M
M
M
M
M
250 -
250 -
180 -
180 -
130 -
130 -
PERK
PARP
95 -
95 -
72 -
72 -
ATF4
55 -
55 -
Actin
eIF2
43 -
43 -
XBP1
34 -
34 -
phospho-eIF2
Casp-3
CHOP
26 -
26 -
17 -
17 -
Cleaved Casp-3
11 -
11 -

## Slide 9
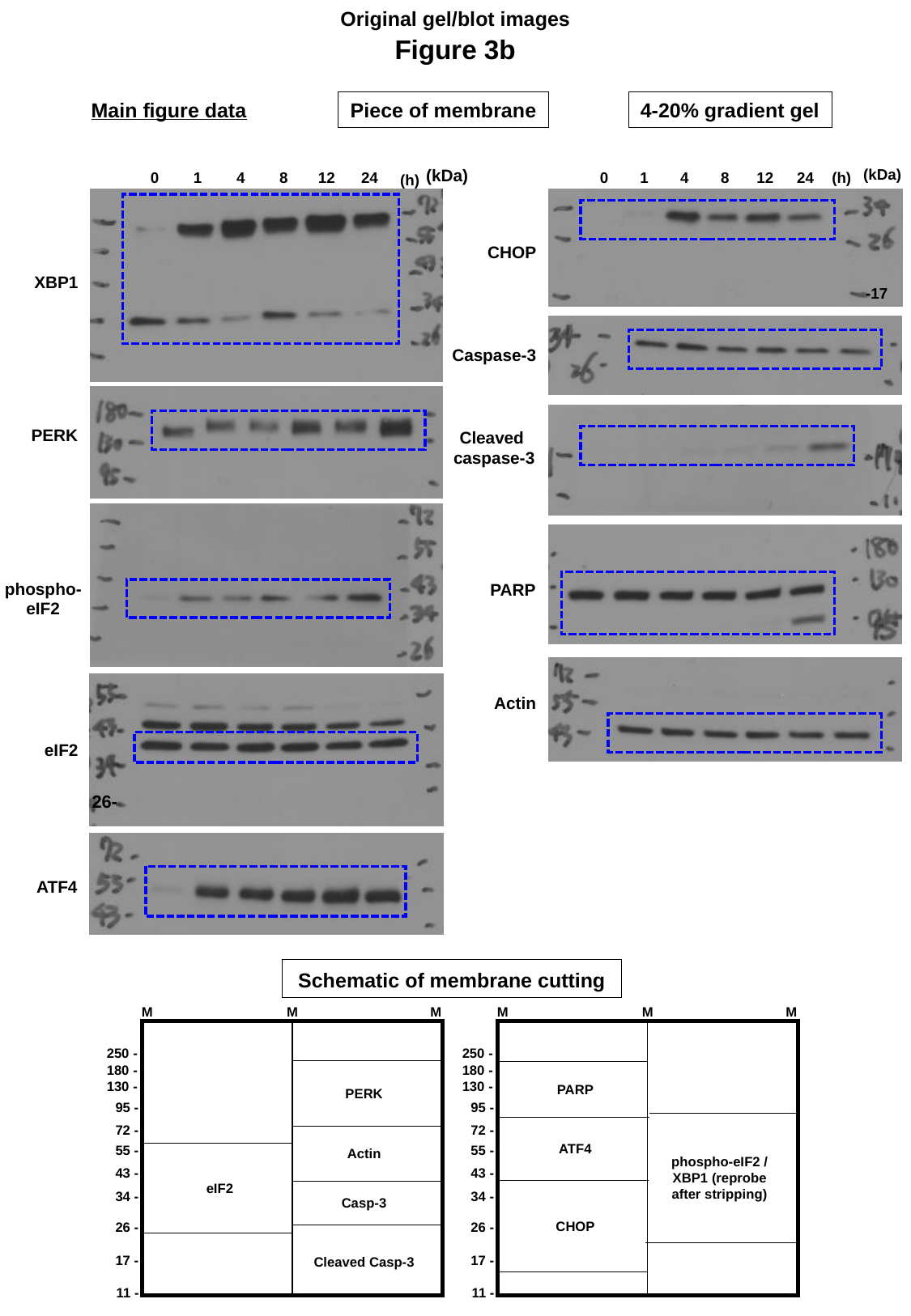

Original gel/blot images
Figure 3b
Main figure data
Piece of membrane
4-20% gradient gel
(kDa)
(kDa)
0
1
4
8
12
24
0
1
4
8
12
24
(h)
(h)
CHOP
XBP1
-17
Caspase-3
PERK
Cleaved
caspase-3
phospho-
eIF2
PARP
Actin
eIF2
26-
ATF4
Schematic of membrane cutting
M
M
M
M
M
M
250 -
250 -
180 -
180 -
130 -
130 -
PARP
PERK
95 -
95 -
72 -
72 -
ATF4
55 -
55 -
Actin
phospho-eIF2 /
XBP1 (reprobe after stripping)
43 -
43 -
eIF2
34 -
34 -
Casp-3
CHOP
26 -
26 -
17 -
17 -
Cleaved Casp-3
11 -
11 -

## Slide 10
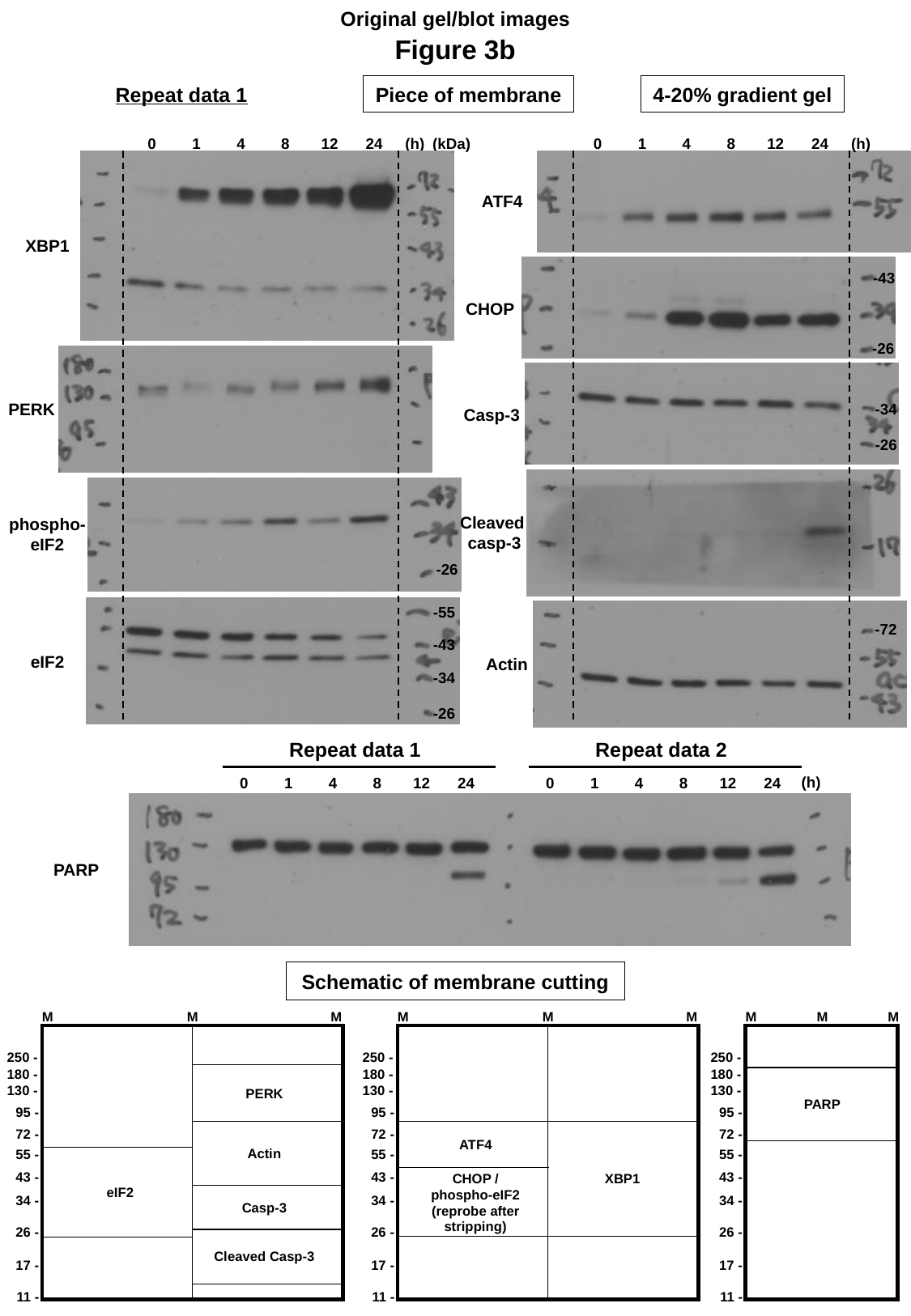

Original gel/blot images
Figure 3b
Repeat data 1
Piece of membrane
4-20% gradient gel
0
1
4
8
12
24
(h)
(kDa)
0
1
4
8
12
24
(h)
ATF4
XBP1
-43
CHOP
-26
PERK
-34
Casp-3
-26
Cleaved
casp-3
phospho-
eIF2
-26
-55
-72
-43
eIF2
Actin
-34
-26
Repeat data 1
Repeat data 2
(h)
0
1
4
8
12
24
0
1
4
8
12
24
PARP
Schematic of membrane cutting
M
M
M
M
M
M
250 -
250 -
180 -
180 -
130 -
130 -
PERK
95 -
95 -
72 -
72 -
ATF4
Actin
55 -
55 -
43 -
43 -
CHOP /
phospho-eIF2
(reprobe after
stripping)
XBP1
eIF2
34 -
34 -
Casp-3
26 -
26 -
Cleaved Casp-3
17 -
17 -
11 -
11 -
M
M
M
250 -
180 -
130 -
PARP
95 -
72 -
55 -
43 -
34 -
26 -
17 -
11 -

## Slide 11
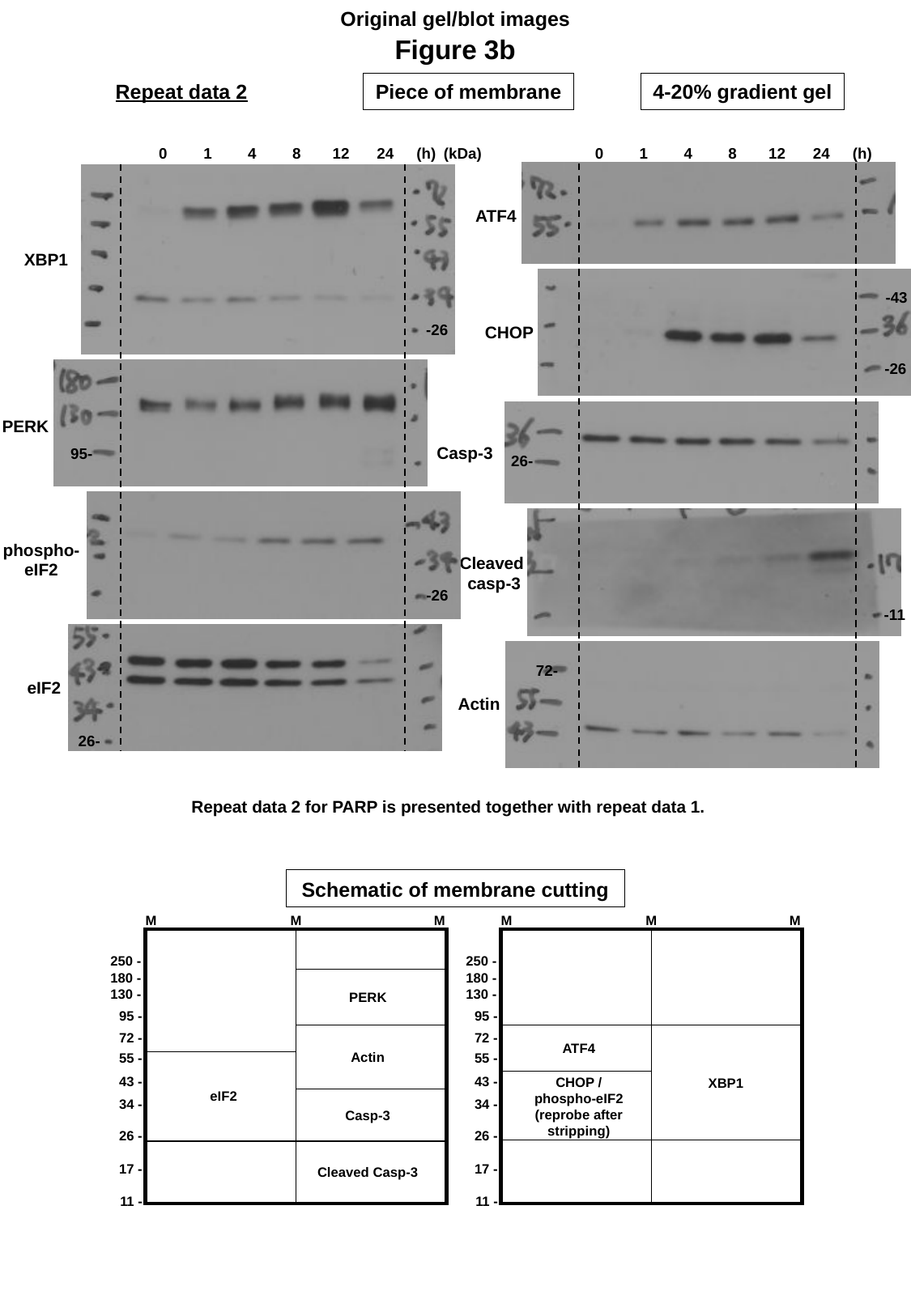

Original gel/blot images
Figure 3b
Repeat data 2
Piece of membrane
4-20% gradient gel
0
1
4
8
12
24
(h)
(kDa)
0
1
4
8
12
24
(h)
ATF4
XBP1
-43
-26
CHOP
-26
PERK
Casp-3
95-
26-
phospho-
eIF2
Cleaved
casp-3
-26
-11
72-
eIF2
Actin
26-
Repeat data 2 for PARP is presented together with repeat data 1.
Schematic of membrane cutting
M
M
M
M
M
M
250 -
250 -
180 -
180 -
130 -
130 -
PERK
95 -
95 -
72 -
72 -
ATF4
Actin
55 -
55 -
43 -
43 -
CHOP /
phospho-eIF2
(reprobe after
stripping)
XBP1
eIF2
34 -
34 -
Casp-3
26 -
26 -
17 -
17 -
Cleaved Casp-3
11 -
11 -

## Slide 12
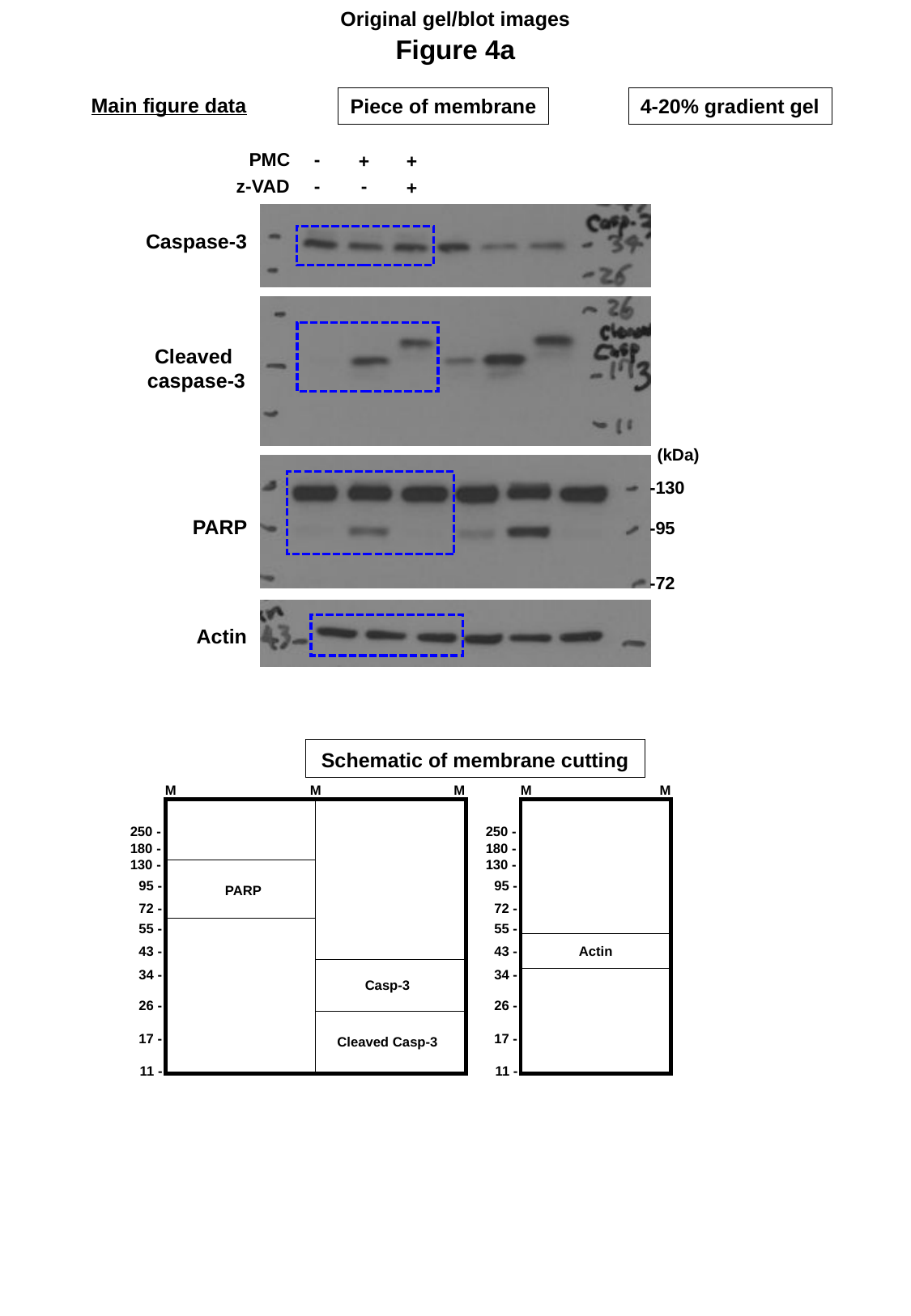

Original gel/blot images
Figure 4a
Main figure data
Piece of membrane
4-20% gradient gel
PMC
-
+
+
z-VAD
-
-
+
Caspase-3
Cleaved
caspase-3
(kDa)
-130
-95
-72
PARP
Actin
Schematic of membrane cutting
M
M
M
M
M
250 -
250 -
180 -
180 -
130 -
130 -
95 -
95 -
PARP
72 -
72 -
55 -
55 -
43 -
43 -
Actin
34 -
34 -
Casp-3
26 -
26 -
17 -
17 -
Cleaved Casp-3
11 -
11 -

## Slide 13
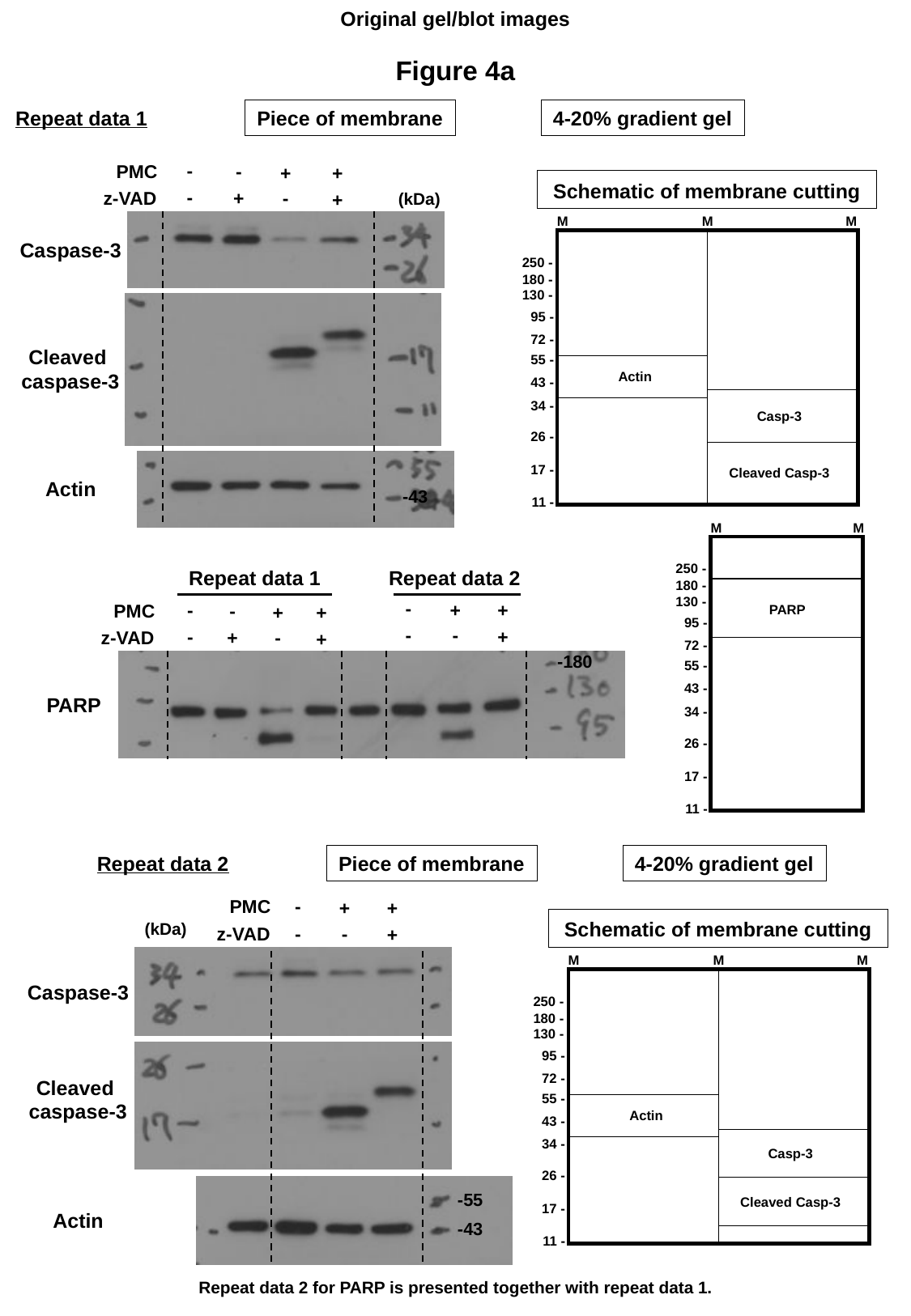

Original gel/blot images
Figure 4a
Repeat data 1
Piece of membrane
4-20% gradient gel
-
PMC
-
+
+
Schematic of membrane cutting
-
z-VAD
+
-
+
(kDa)
M
M
M
250 -
180 -
130 -
95 -
72 -
55 -
Actin
43 -
34 -
Casp-3
26 -
17 -
Cleaved Casp-3
11 -
Caspase-3
Cleaved
caspase-3
Actin
-43
M
M
250 -
180 -
130 -
PARP
95 -
72 -
55 -
43 -
34 -
26 -
17 -
11 -
Repeat data 1
Repeat data 2
-
-
+
+
PMC
-
+
+
-
-
-
+
z-VAD
+
-
+
-180
PARP
Repeat data 2
Piece of membrane
4-20% gradient gel
PMC
-
+
+
Schematic of membrane cutting
(kDa)
z-VAD
-
-
+
M
M
M
250 -
180 -
130 -
95 -
72 -
55 -
Actin
43 -
34 -
Casp-3
26 -
Cleaved Casp-3
17 -
11 -
Caspase-3
Cleaved
caspase-3
-55
Actin
-43
Repeat data 2 for PARP is presented together with repeat data 1.

## Slide 14
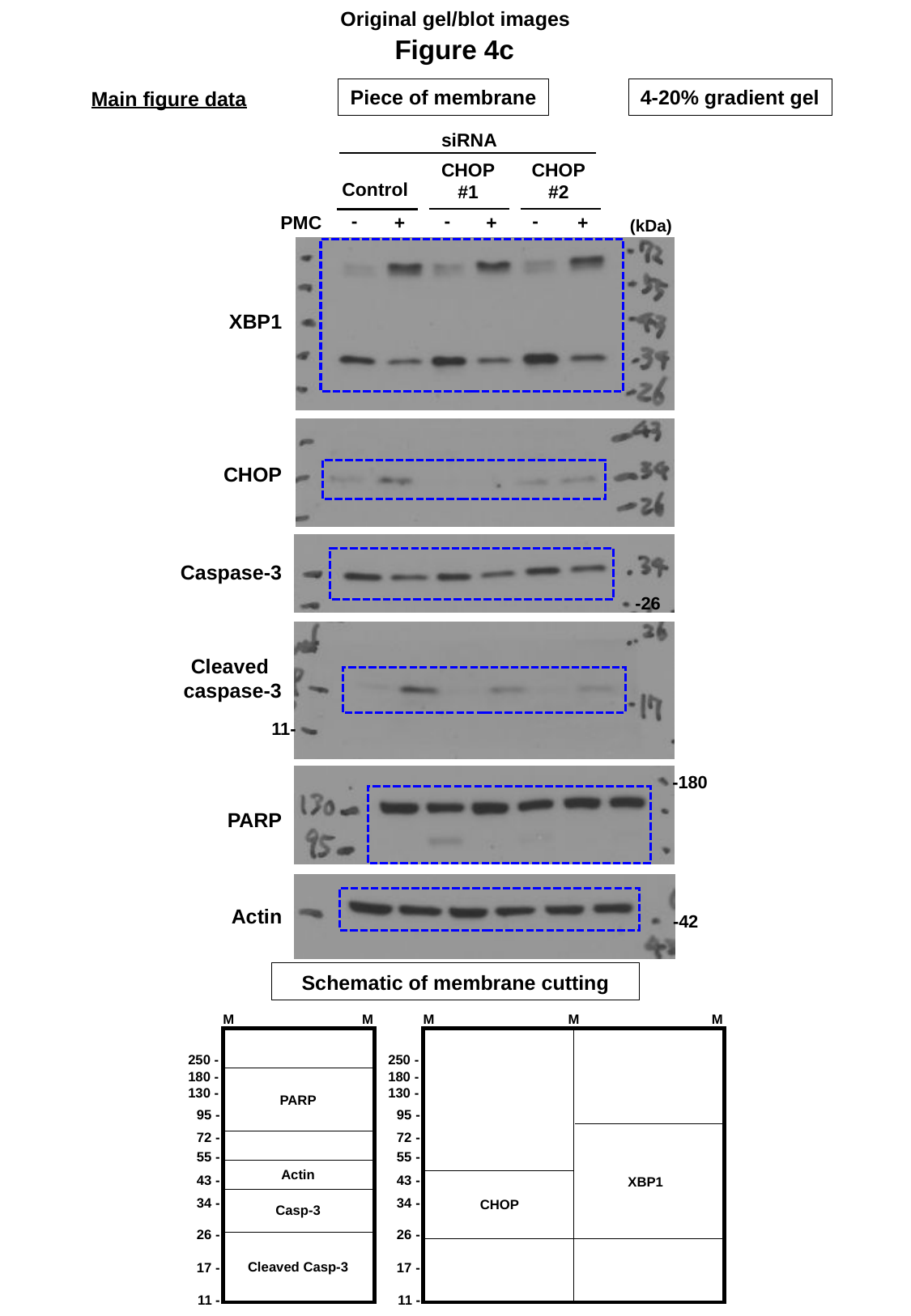

Original gel/blot images
Figure 4c
Piece of membrane
4-20% gradient gel
Main figure data
siRNA
CHOP
#2
CHOP
#1
Control
-
-
-
+
+
+
PMC
(kDa)
XBP1
CHOP
Caspase-3
-26
11-
Cleaved
caspase-3
-180
PARP
Actin
-42
Schematic of membrane cutting
M
M
M
M
M
250 -
250 -
180 -
180 -
130 -
130 -
PARP
95 -
95 -
72 -
72 -
55 -
55 -
Actin
43 -
43 -
XBP1
34 -
34 -
CHOP
Casp-3
26 -
26 -
Cleaved Casp-3
17 -
17 -
11 -
11 -

## Slide 15
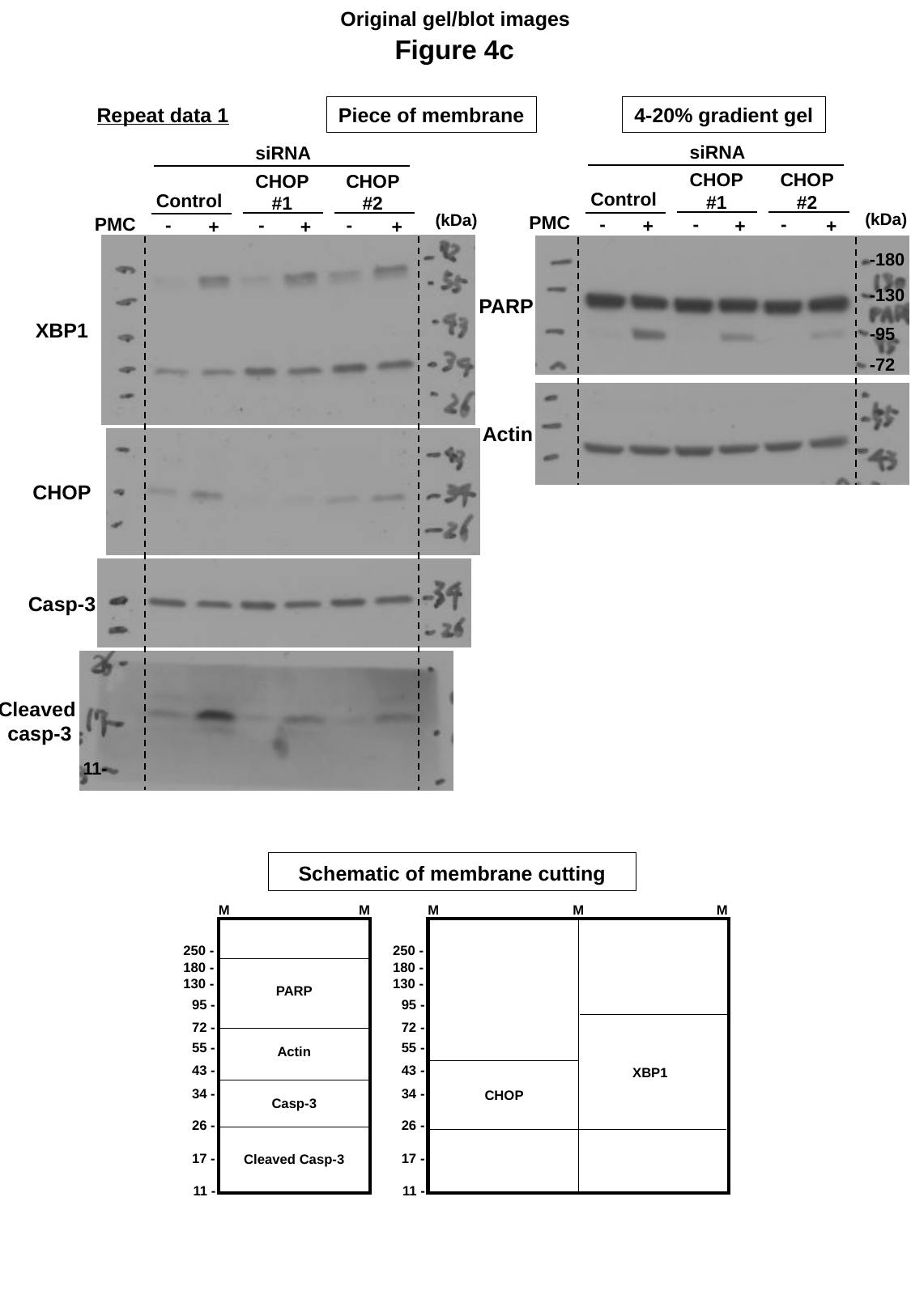

Original gel/blot images
Figure 4c
Repeat data 1
Piece of membrane
4-20% gradient gel
siRNA
siRNA
CHOP
#2
CHOP
#1
CHOP
#2
CHOP
#1
Control
Control
(kDa)
(kDa)
PMC
PMC
-
-
-
-
-
-
+
+
+
+
+
+
-180
-130
PARP
XBP1
-95
-72
Actin
CHOP
Casp-3
Cleaved
casp-3
11-
Schematic of membrane cutting
M
M
M
M
M
250 -
250 -
180 -
180 -
130 -
130 -
PARP
95 -
95 -
72 -
72 -
55 -
55 -
Actin
43 -
43 -
XBP1
34 -
34 -
CHOP
Casp-3
26 -
26 -
17 -
17 -
Cleaved Casp-3
11 -
11 -

## Slide 16
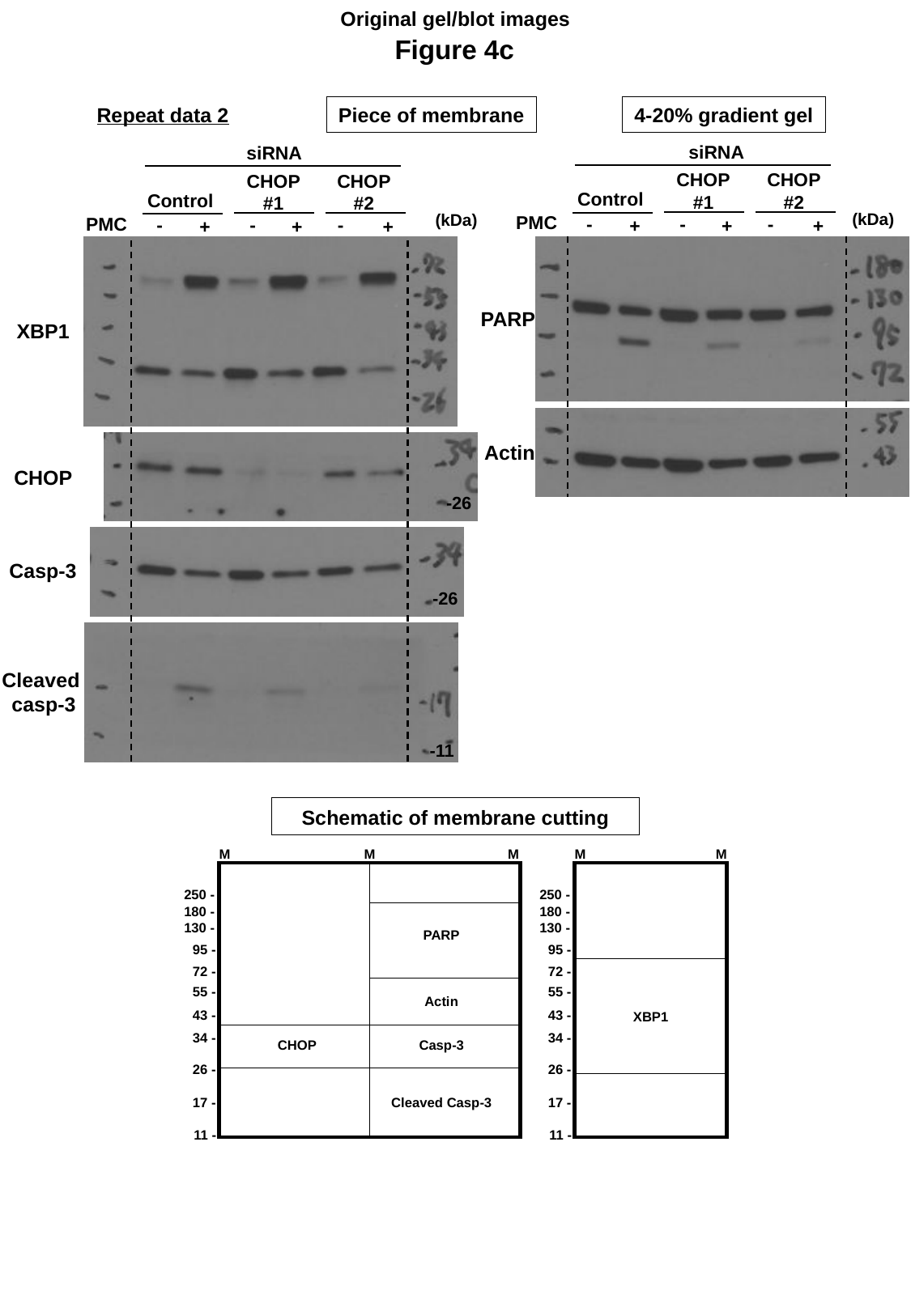

Original gel/blot images
Figure 4c
Repeat data 2
Piece of membrane
4-20% gradient gel
siRNA
siRNA
CHOP
#2
CHOP
#1
CHOP
#2
CHOP
#1
Control
Control
(kDa)
(kDa)
PMC
PMC
-
-
-
-
-
-
+
+
+
+
+
+
PARP
XBP1
Actin
CHOP
-26
Casp-3
-26
Cleaved
casp-3
-11
Schematic of membrane cutting
M
M
M
M
M
250 -
250 -
180 -
180 -
130 -
130 -
PARP
95 -
95 -
72 -
72 -
55 -
55 -
Actin
43 -
43 -
XBP1
34 -
34 -
Casp-3
CHOP
26 -
26 -
Cleaved Casp-3
17 -
17 -
11 -
11 -

## Slide 17
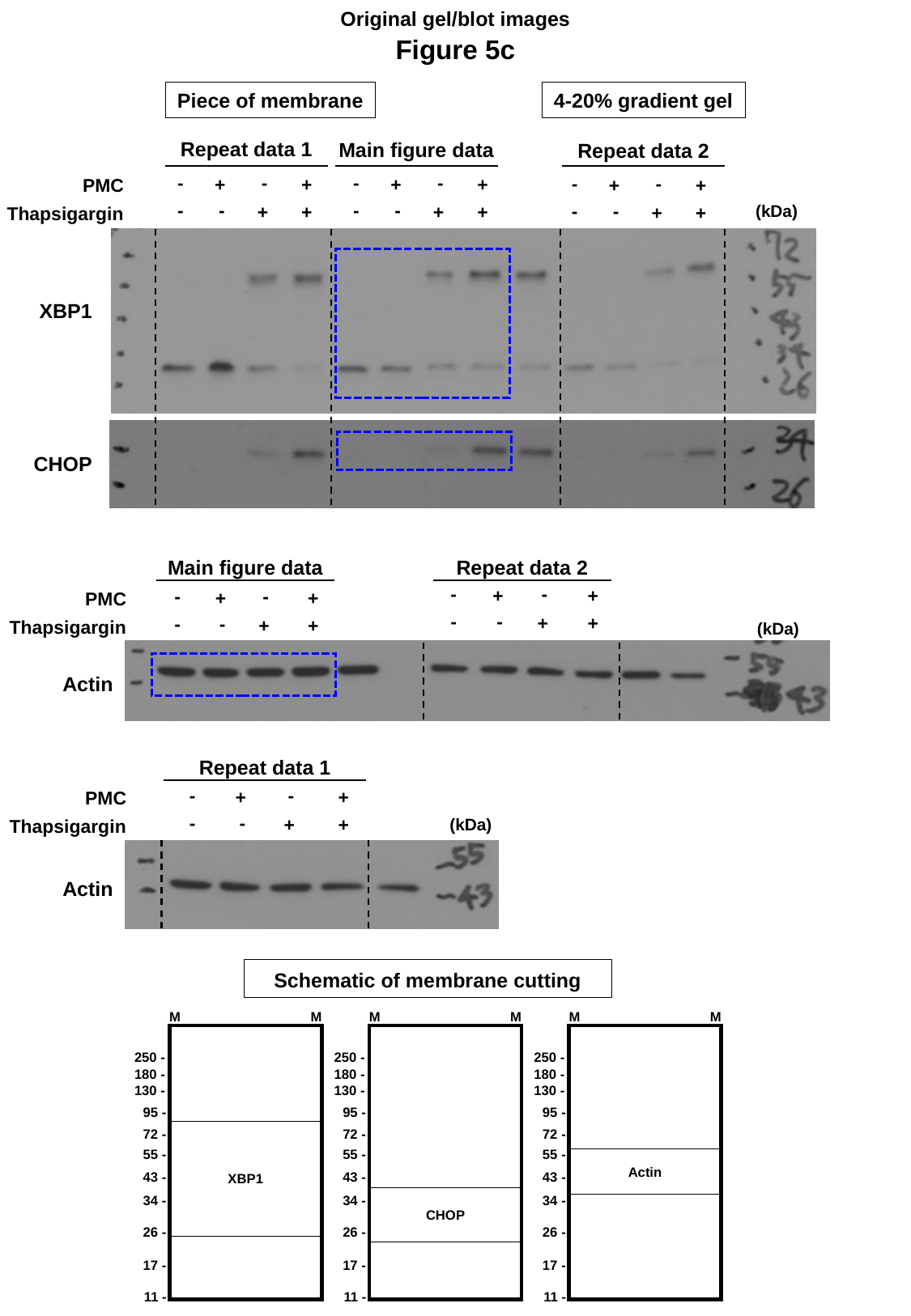

Original gel/blot images
Figure 5c
Piece of membrane
4-20% gradient gel
Repeat data 1
Main figure data
Repeat data 2
-
-
-
-
-
-
+
+
+
+
PMC
+
+
-
-
-
-
-
-
+
+
+
+
(kDa)
+
+
Thapsigargin
XBP1
CHOP
Main figure data
Repeat data 2
-
-
+
+
-
-
+
+
PMC
-
-
+
+
-
-
+
+
Thapsigargin
(kDa)
Actin
Repeat data 1
-
-
+
+
PMC
-
-
+
+
(kDa)
Thapsigargin
Actin
Schematic of membrane cutting
M
M
M
M
M
M
250 -
250 -
250 -
180 -
180 -
180 -
130 -
130 -
130 -
95 -
95 -
95 -
72 -
72 -
72 -
55 -
55 -
55 -
Actin
43 -
43 -
43 -
XBP1
34 -
34 -
34 -
CHOP
26 -
26 -
26 -
17 -
17 -
17 -
11 -
11 -
11 -

## Slide 18
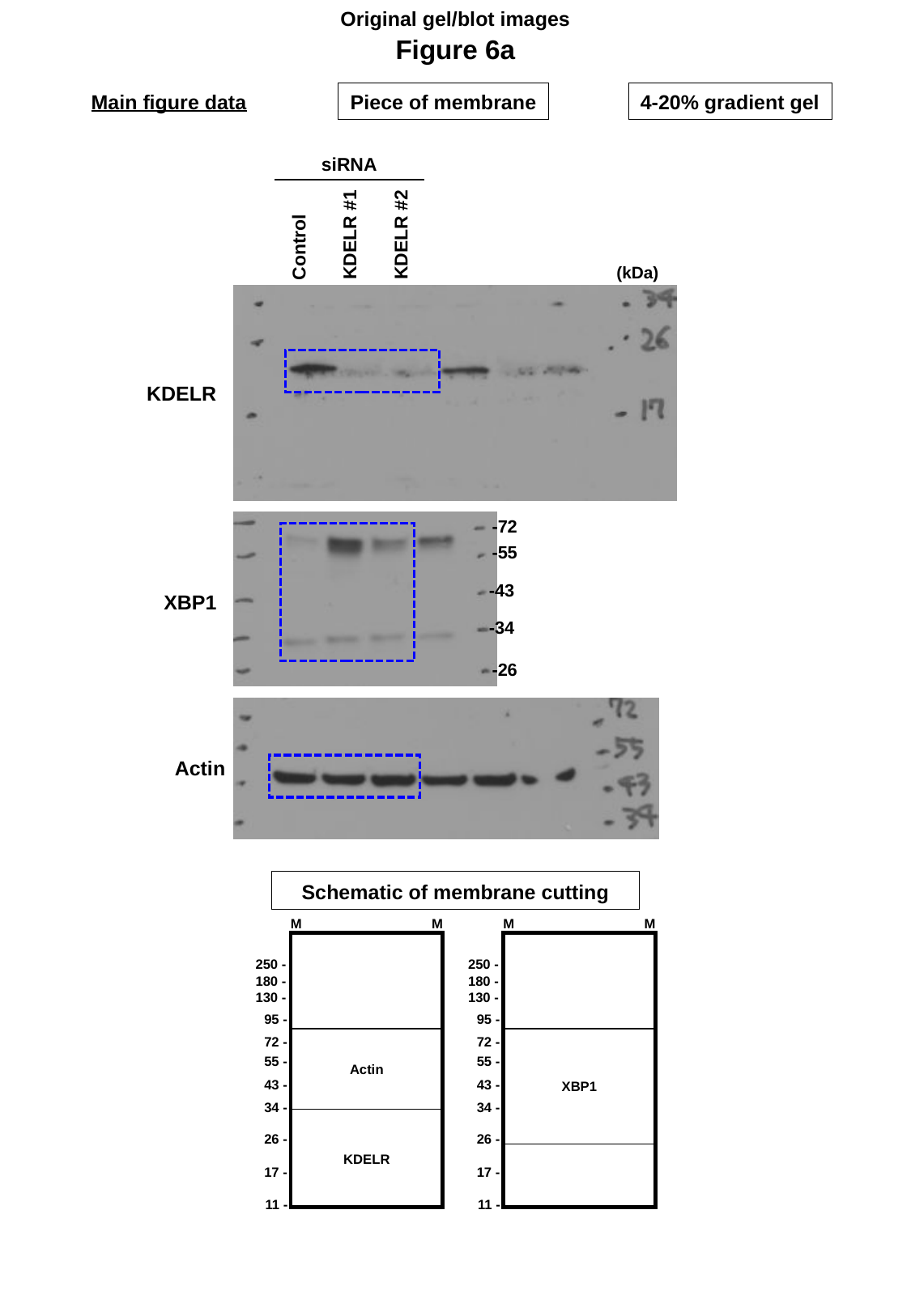

Original gel/blot images
Figure 6a
Main figure data
Piece of membrane
4-20% gradient gel
siRNA
KDELR #1
KDELR #2
Control
(kDa)
KDELR
-72
-55
-43
-34
-26
XBP1
Actin
Schematic of membrane cutting
M
M
250 -
180 -
130 -
95 -
72 -
55 -
Actin
43 -
34 -
26 -
KDELR
17 -
11 -
M
M
250 -
180 -
130 -
95 -
72 -
55 -
43 -
XBP1
34 -
26 -
17 -
11 -

## Slide 19
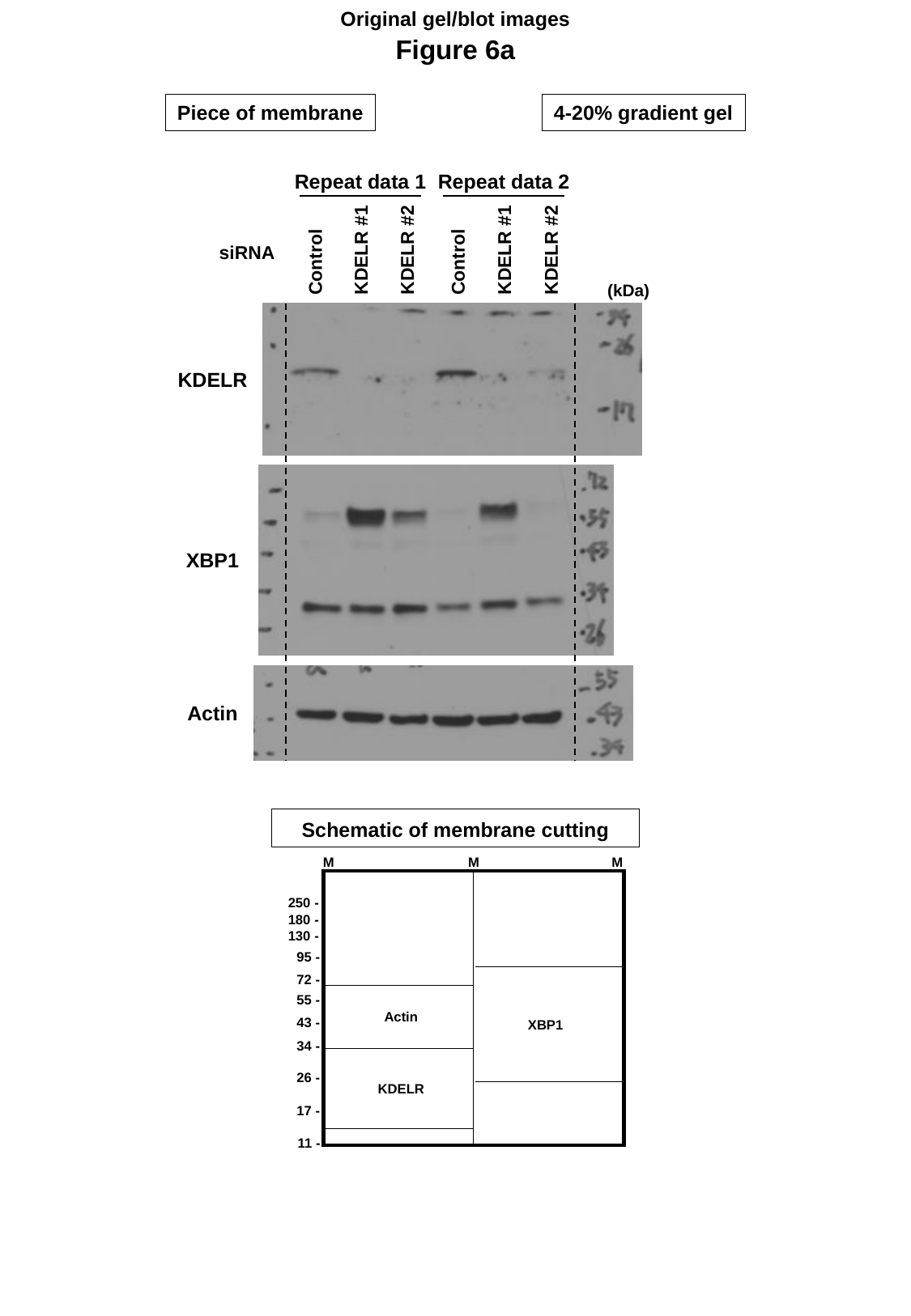

Original gel/blot images
Figure 6a
Piece of membrane
4-20% gradient gel
Repeat data 1
Repeat data 2
KDELR #1
KDELR #2
KDELR #1
KDELR #2
siRNA
Control
Control
(kDa)
KDELR
XBP1
Actin
Schematic of membrane cutting
M
M
M
250 -
180 -
130 -
95 -
72 -
55 -
Actin
43 -
XBP1
34 -
26 -
KDELR
17 -
11 -
